# Modeling acute myocardial infarction and cardiac fibrosis using human induced pluripotent stem cell-derived multi-cellular heart organoids

**DOI:** 10.1038/s41419-024-06703-9

**Published:** 2024-05-01

**Authors:** Myeongjin Song, Da Bin Choi, Jeong Suk Im, Ye Na Song, Ji Hyun Kim, Hanbyeol Lee, Jieun An, Ami Kim, Hwan Choi, Joon-Chul Kim, Choongseong Han, Young Keul Jeon, Sung Joon Kim, Dong-Hun Woo

**Affiliations:** 1 Department of Commercializing Organoid Technology, NEXEL Co., Ltd., Seoul, 07802 Korea; 2 Centre for Research, Hudson Institute of Medical Research, Monash University, Clayton, VIC 3168 Australia; 3 Department of Commercializing iPSC Technology, NEXEL Co., Ltd., Seoul, 07802 Korea; 4 Department of Physiology, Seoul National University College of Medicine, Seoul, 03080 Korea; 5 Ischemic/Hypoxic Disease Institute, Seoul National University, College of Medicine, Seoul, 03080 Korea

**Keywords:** Diseases, Stem-cell biotechnology, Induced pluripotent stem cells

## Abstract

Heart disease involves irreversible myocardial injury that leads to high morbidity and mortality rates. Numerous cell-based cardiac in vitro models have been proposed as complementary approaches to non-clinical animal research. However, most of these approaches struggle to accurately replicate adult human heart conditions, such as myocardial infarction and ventricular remodeling pathology. The intricate interplay between various cell types within the adult heart, including cardiomyocytes, fibroblasts, and endothelial cells, contributes to the complexity of most heart diseases. Consequently, the mechanisms behind heart disease induction cannot be attributed to a single-cell type. Thus, the use of multi-cellular models becomes essential for creating clinically relevant in vitro cell models. This study focuses on generating self-organizing heart organoids (HOs) using human-induced pluripotent stem cells (hiPSCs). These organoids consist of cardiomyocytes, fibroblasts, and endothelial cells, mimicking the cellular composition of the human heart. The multi-cellular composition of HOs was confirmed through various techniques, including immunohistochemistry, flow cytometry, q-PCR, and single-cell RNA sequencing. Subsequently, HOs were subjected to hypoxia-induced ischemia and ischemia-reperfusion (IR) injuries within controlled culture conditions. The resulting phenotypes resembled those of acute myocardial infarction (AMI), characterized by cardiac cell death, biomarker secretion, functional deficits, alterations in calcium ion handling, and changes in beating properties. Additionally, the HOs subjected to IR efficiently exhibited cardiac fibrosis, displaying collagen deposition, disrupted calcium ion handling, and electrophysiological anomalies that emulate heart disease. These findings hold significant implications for the advancement of in vivo-like 3D heart and disease modeling. These disease models present a promising alternative to animal experimentation for studying cardiac diseases, and they also serve as a platform for drug screening to identify potential therapeutic targets.

## Introduction

Organoids are distinctive three-dimensional (3D) cellular models, as they effectively recapitulate numerous aspects of the intricate structure and functionality observed in tissues, making them invaluable tools in biological and medical research [1].

Unlike other tissues, the heart has limited capacity for regeneration, and endomyocardial biopsies are difficult to obtain [2–4]. Consequently, many researchers have endeavored to generate 3D cardiac models from induced pluripotent stem cells (iPSCs) to facilitate in vitro cardiac studies [2, 5]. However, the most previously reported cardiac organoid (CO) models center on a 3D architecture constructed via the cardiac spheroid technique, which entails fusing iPSC-derived cardiomyocytes [6, 7].

Conversely, several studies have unveiled COs encompassing diverse cell populations. Drakhils et al., for instance, harnessed human iPSC aggregates embedded in matrigel to generate human heart-forming organoids, thereby recapitulating early human heart and foregut development [8]. Similarly, another research group generated iPSC-derived cardioids harboring key cardiac cell types [9, 10]. These cardioids exhibited the development of small and large cavities mirroring heart maturation [11]. However, while these cardiac models are suitable for studying early heart development, there remains a critical need for the development of more robust 3D cardiac models that faithfully mimic the intricate attributes of adult heart tissue with multi-cellular compositions.

CO models also have the potential to significantly advance studies related to human physiological functions of tissues and the pathological mechanisms underlying human diseases [12, 13]. These models address limitations posed by animal models and traditional 2D monolayer cell cultures, which fail to replicate certain aspects of human biology [14, 15]. In a recent study, localized cryoinjury induced extracellular matrix accumulation in COs [11, 16]. Another study involved culturing iPSC-derived COs with 10% O_2_ and 1 μM noradrenaline, creating an apoptosis gradient that emulates the myocardial infarction environment [17]. These reports shed light on molecular and cellular regulatory mechanisms governing the regenerative capacity of immature human hearts and establish an in vitro model for cardiovascular disease. Nonetheless, these organoids have struggled to comprehensively mimic adult human heart failure, encompassing the intricate mechanisms of the disease.

In this study, our focus was on developing multi-cellular heart organoids (HOs) from human iPSCs mirroring the proportional composition of the human adult heart. Subsequently, perturbation of cellular interactions in the HOs by defined culture conditions efficiently recapitulated the heart disease from initial responses following myocardial infarction to subsequent fibrotic activation in the HOs.

## Materials and methods

### hiPSCs culture and generation of COs and HOs

The hiPSCs were expanded and cultured under feeder-free conditions on Matrigel (Corning, USA) coated T75 tissue culture flasks in the presence of mTeSR1 (Stem Cell Technologies, Canada). Cardiac organoid (CO) differentiation was performed as described in our previous reports [18]. To differentiate heart organoids (HOs), we initially applied the differentiation procedures used for COs to induce mesodermal differentiation. However, the differentiated mesoderm was subsequently cultured with BMP, VEGF, FGF, and TGF-beta-containing medium from D8 to D14 to induce differentiation into multi-lineages. Following multi-lineage induction, the HOs were maintained in RPMI 1640 medium supplemented with 10 ng/ml of TGFβ and B-27 without vitamin A until day 30 (D30).

Further details on the induction of disease models and analysis were described in the supplementary information.

## Results

### Derivation of HOs from hiPSCs

**Fig. 1:**
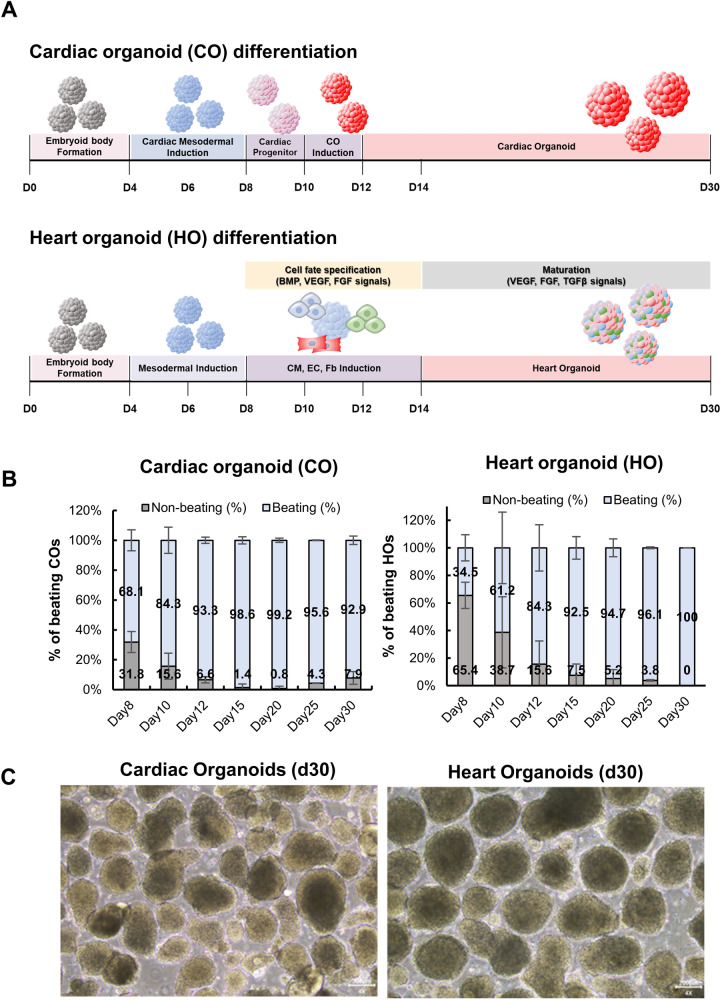
Generation of hiPSC-derived cardiac organoids (COs) and heart organoids (HOs). **A** Overall schematic diagram of differentiation from hiPSC into COs and HOs. **B** Comparison of beating efficiency of COs and HOs at day 8–30 of differentiation. **C** The morphology of COs and HOs for 30 days, including the differentiation period.

We previously generated hiPSC-derived cardiac organoids (COs), and the CO formation resulted in the enhanced maturity of hiPSC-derived cardiomyocytes [18]. However, these COs could not mimic the diverse cellular composition of the human heart. Thus, we refined our differentiation protocol to generate heart organoids (HOs) designed to accommodate the coexistence of various cardiac lineage cells by modulating BMP, VEGF, FGF, and TGFβ signaling during differentiation (Fig. 1A).

An assessment of organoid beating efficiency spanned the entire differentiation period from day 8 to day 30 for both COs and HOs (Fig. 1B). On day 8–10 of differentiation, both COs and HOs initiated discernible beating (Fig. 1B). Notably, COs showed a beating efficiency of 68.1%, whereas HOs exhibited a lower rate of 34.5% on day 8 of differentiation. However, although the beating efficiency at the beginning of differentiation was higher in COs than in HOs, HOs displayed an ascending trend in their beating rate post-differentiation initiation, culminating in a 100% beating efficiency by day 30 (Fig. 1B), and resulted in similar beating efficiency to COs on day 30 (Fig. 1C, Videos 1 and 2).

Moreover, we validated the cardiomyocyte subtypes in the organoids by confirming the expression levels of MLC-2v and MLC-2a, indicative of ventricular and atrial types of cardiomyocytes, respectively. We observed that the expression of MLC-2a initiated early in differentiation (from D5 to D10), while MLC-2v expression gradually increased over the differentiation period. At D24, the majority of cardiomyocytes in both COs and HOs exhibited strong expression of MLC-2v (Fig. S1A and B).

### Cellular compositions of HOs

**Fig. 2:**
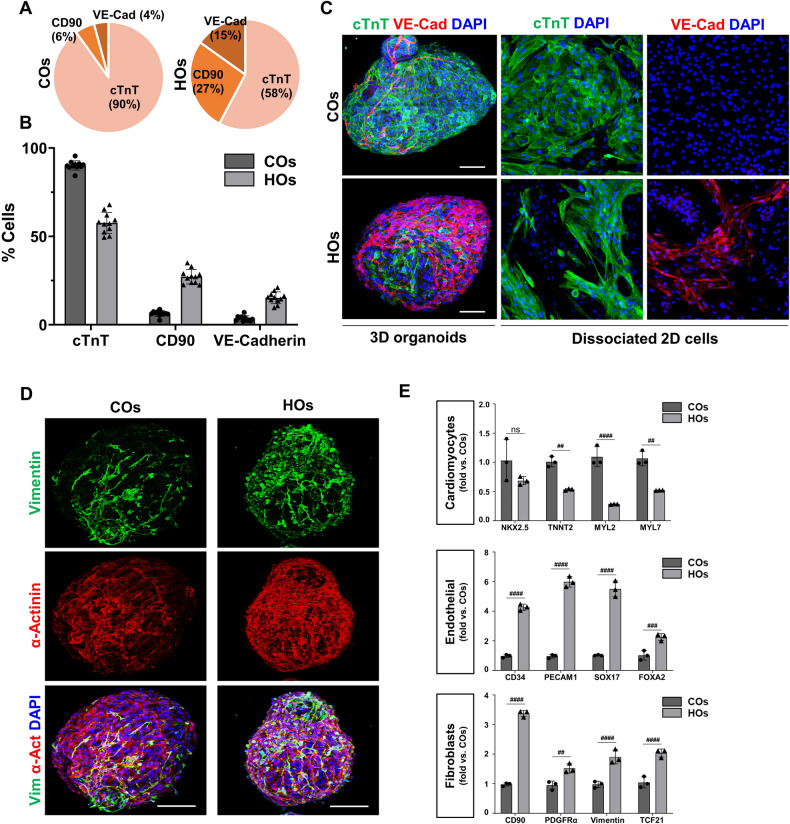
Phenotypical comparison of iPSC-derived COs and HOs. **A** Representative pie chart showing the distribution of cTnT, a cardiomyocyte (CM)-specific marker, CD90, a cardiac fibroblast (CF)-specific marker, and VE-Cad, an endothelial cell (EC)-specific marker, in CO and HO. **B** The graph displays the mean cellular compositions of cardiomyocytes, fibroblasts, and endothelial cells across 11 different batches of COs and HOs. **C** Representative z-stack image of HOs and COs using confocal microscopy (left). Staining for cTnT (green) and VE-cad (red) in 2D monolayer culture of cells dissociated from HOs and COs (right). Scale bar: 100 μm. **D** Z-stack image of co-staining for Vimentin (fibroblast marker, green), α-actinin (cardiomyocyte marker, red), and DAPI (blue) in COs and HOs. Scale bar: 100 μm. **E** Comparison of the gene expression levels for various cell types in COs and HOs. Quantitative analysis of gene expression levels as performed with real-time PCR. The expression levels of cardiomyocyte markers (NKX2.5, TNNT2, MYL2, and MYL7), endothelial cell markers (CD34, PECAM1, SOX17, and FOXA2), fibroblast markers (CD90, PDGFRα, Vimentin, and TCF21) normalized to that of GAPDH. Data were shown as fold-change relative to COs, as mean ± SD, by 2-way ANOVA (*n* = 3). A significant difference is indicated by ^#^*p* < 0.05, ^##^*p* < 0.01, ^###^*p* < 0.001, ^####^*p* < 0.0001 compared with COs and ns (non-significant).

To assess the cellular distribution in the COs and HOs, FACS analysis was performed (Figs. 2A, B and S2). Within COs, the mean distribution of cTnT, CD90, and VE-cadherin was 92.23 ± 2.62%, 6.24 ± 1.55%, and 4.73 ± 1.48%, respectively (Figs. 2A, B and S2). Conversely, in HOs, these distributions were determined to be 51.09 ± 5.92%, 24.63 ± 4.08%, and 14.03 ± 3.34%, respectively (Figs. 2A, B and S2).

Immunostaining was also performed to visualize the distribution of cardiac lineage cells (Fig. 2C and D). Consistently with FACS analysis, COs prominently displayed cTnT expression within the organoids, while HOs displayed VE-cadherin expression on the surface of HOs (Fig. 2C, Videos 3, 4). The distinct distribution of blood vessel cells in HOs was further verified through the culture of dissociated organoids, and the dissociated cells from HOs exhibited a composition of both cTnT-positive cells and VE-Cad-positive cells (Fig. 2C). In contrast, the dissociated cells from COs exclusively displayed cTnT-positive cells (Fig. 2C). In addition, the HOs exhibited notable expression of Vimentin, a fibroblast marker, in comparison to COs (Fig. 2D).

The differences were further validated through qPCR analysis, and this analysis substantiated that COs exhibited a relatively increased expression of NKX 2.5, a transcription factor governing heart development, in addition to TNNT2, MYL2, and MYL7 (Fig. 2E). In contrast, HOs displayed significant upregulation in genes related to cardiac fibroblasts (CD90 and PDGFRα), Vimentin, and TCF21, all in comparison to COs (Fig. 2E). Furthermore, endothelial-related genes including CD34, PECAM1, SOX17, and FOXA2 exhibited elevated expression levels in HOs relative to COs (Fig. 2E).

### Single-cell transcriptomic analysis of HOs

**Fig. 3:**
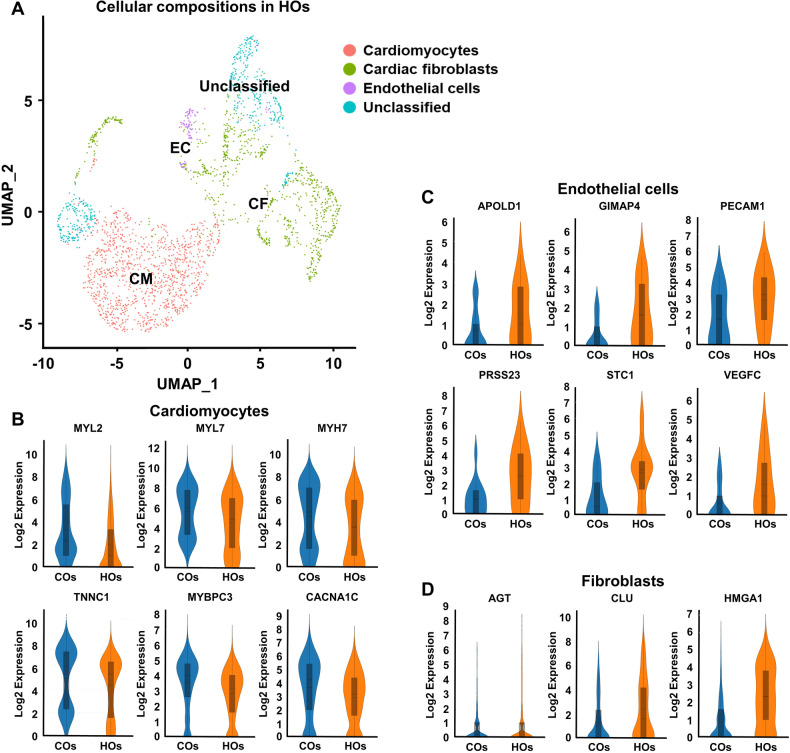
Single-cell RNA-sequencing analysis of COs and HOs. **A** Uniform manifold approximation and projection (UMAP) plots of HOs. Winseurat data sets labeled with Winseurat clusters. Detailed clustering within CMs (pink), CFs (green), and ECs (purple) clusters. **B**–**D** Violin plots of representative genes for CMs (MYL2, MYL7, MYH7, TNNC1, MYBPC3, and CACNA1C), ECs (APOLD1, GIMAP4, PECAM1, PRSS23, STC1, and VEGFC), and CFs (AGT, CLU, and HMGA1). These genes were selected fold-change about 2-fold, average expression about 4 or more, and *p*-value 0.05 or less.

To gain a deeper understanding of the intricate gene expression within HOs, we utilized single-cell RNA sequencing (Fig. 3). Utilizing UMAP clustering and marker identification, the pool of 2587 cells from HOs was effectively categorized into distinct groups, encompassing cardiomyocytes, fibroblasts, and endothelial cells (Fig. 3A). To compare the representative gene expression patterns by cell type in both COs and HOs, we selected genes with *p*-values below 0.05 and visualized their expression using violin plots. Additionally, a comparative examination of gene expression patterns between COs and HOs was presented through violin plots. Notably, representative genes linked to cardiomyocytes, such as MYL2, MYL7, MYH7, TNNC1, MYBP3, and CACNA1C, exhibited enhanced expression in COs when compared to HOs (Fig. 3B). Similarly, an analysis was also extended to endothelial cells. Endothelial-related genes such as APOLD1, GIMAP4, PECAM1, PRSS23, STC1, and VEGFC exhibited an upregulation in HOs compared to COs (Fig. 3C). Shifting the focus to cardiac fibroblasts, the violin plots highlighted genes like AGT, CLU, and HMGA1 illustrating disparities between COs and HOs (Fig. 3D).

Clusters where unselected genes were grouped under the “unclassified” category in HOs were further analyzed (Fig. 3A). To predict cell types within the populations, we made use of databases such as pangiaoDB and GeneCard. These predictions revealed that the unclassified population mainly comprised pericytes, epithelial cells, neurons, and other cell types. These findings collectively indicate that HOs possess a more comprehensive genetic repertoire of heart constituent cells in comparison to COs.

### Increased sensitivity to ischemic injury and ischemic-reperfusion (IR) injury in HOs

**Fig. 4:**
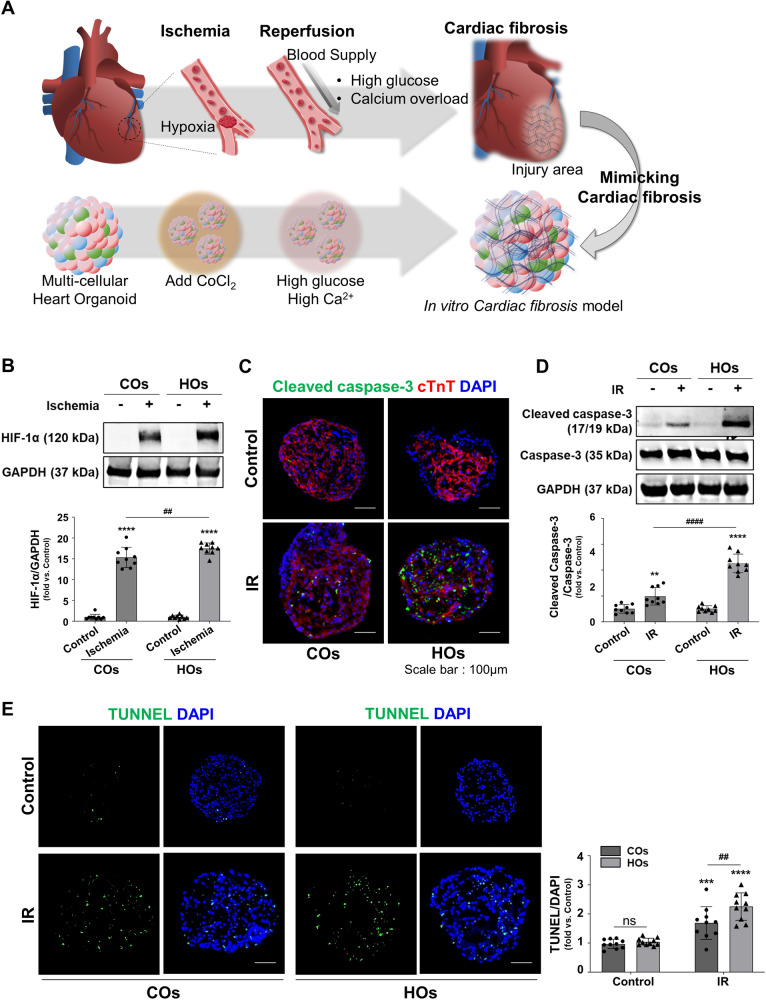
Induction of ischemic injury and ischemic-reperfusion injury in COs and HOs. **A** Schematic of an experiment mimicking the heart disease in organoids by ischemia-reperfusion injury mechanism that occurs in the human adult heart and followed fibrogenesis. **B** Expression of the HIF-1α through western blots. Quantitative analysis of HIF-1α performed using Image J software. The expression of HIF-1α normalized to that of GAPDH. Data were shown as fold-change, and a significant difference is indicated by ****^, ####^*p* < 0.0001, and ns (non-significant). **C** Immunofluorescence images of the expression levels of apoptotic marker (cleaved caspase-3, green) and cardiomyocyte marker (cTnT, red) in COs and HOs after IR injury. The scale bar represents 100 μm. **D** Representative western blot image and quantitative analysis of cleaved caspase-3. Data normalized to that of caspase-3. Equal protein loading amounts were confirmed by GAPDH expression. The corresponding density ratio was calculated by the average intensity of the bands from Image J software. Data were shown as fold-change, and a significant difference is indicated by ****^, ####^*p* < 0.0001 and ns (non-significant). **E** Representative image and quantitative analysis of TUNEL assay (green). Data were shown as fold-change, as mean ± SD, by 2-way ANOVA (*n* = 3). Significant difference is indicated by ^#^*p* < 0.05, ***p < 0.001^,^ ****^, ####^*p* < 0.0001 (*compared to control group; ^#^com*p*ared to COs), and ns (non-significant). The scale bar represents 100 μm.

With the established HOs, we mimicked the pathological conditions of acute myocardial infarction (AMI) and subsequent cardiac fibrosis to model human heart disease (Fig. 4A). To replicate hypoxia-induced ischemic conditions, we introduced 50 μM of cobalt chloride (CoCl_2_) [19] to the organoids along with glucose-depleted culture medium. This approach effectively elevated the expression of hypoxia-inducible factor-1α (HIF-1α) in both COs and HOs (Figs. 4B and S3).

In the clinical condition, the rapid reintroduction of blood flow post-reperfusion can lead to an immediate supply of oxygen and nutrients, triggering heightened inflammation and oxidative stress, thereby potentially causing tissue damage. A study suggested that high glucose sensitizes cardiomyocytes to ischemia-reperfusion (IR) injury [20]. Another study proposed that intracellular and mitochondrial calcium overload may contribute to reperfusion injury by exacerbating oxidative stress [21]. Based on these findings, we hypothesized that these factors could mimic reperfusion injury in CoCl_2_-treated HOs. To test this hypothesis, we applied a culture condition in which high glucose and calcium ion levels, then CoCl_2_-treated COs and HOs were exposed to a reperfusion medium rich in glucose and calcium ions for 72 h.

To verify the induction of apoptosis in both COs and HOs following IR injury, we performed co-staining of cTnT and cleaved caspase-3 in organoid sections (Fig. 4C). This co-staining provided confirmation of the reduction in cardiomyocytes and the increase in apoptosis were more pronounced in HOs compared to COs (Fig. 4C). Western blot analysis against the cleaved caspase-3 further confirmed the more effective induction of apoptosis in HOs relative to COs (Figs. 4D and S4). Validation of apoptosis in the IR-injured organoids was also carried out using the TUNEL assay, and the findings demonstrated the more increased apoptotic cells within the HOs than that of COs (Fig. 4E). Additionally, Western blot analysis unveiled a more substantial increase in the Bax/Bcl2 signaling in HOs compared to COs (Fig. S5).

### IR-injured HOs exhibited characteristics of human AMI

IR injury in humans leads to the disruption of sarcomere structures and a reduction in cardiac markers, including cTnT and cTnI, within heart tissue [22]. Furthermore, in clinical practices, markers such as cTnI, Myoglobin (MB), and Creatine kinase M (CKM) are quantified in blood to diagnose myocardial infarction resulting from IR injury [23].

**Fig. 5:**
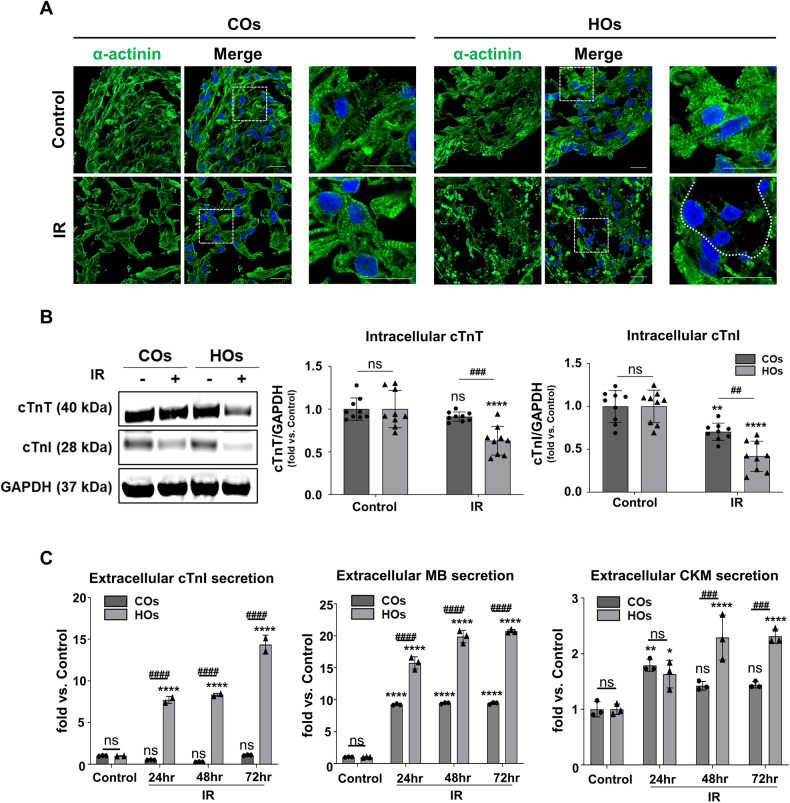
IR-induced acute myocardial infarction (AMI) in COs and HOs. **A** Immunofluorescence images of the expression levels of sarcomeric α-actinin and DAPI in the control and IR groups. White dotted line in IR-induced HOs indicates the disintegrated sarcomere structure in the organoids. The scale bar represents 100 μm and magnified image scale bar represents 20 μm. **B** Western blot analysis in cell lysates from COs and HOs in control and IR groups. The protein expression of cTnT and cTnI, which are essential for cardiac structure was calculated by the average intensity of the bands from Image J software. The comparison of the fold change between the COs and HOs was normalized by the control group. **C** Extracellular secretion levels of cTnI, myoglobin (MB), and creatine kinase M type (CKM), which AMI indicators in control and IR group during culture periods. Secretion of cTnI, MB, and CKM was measured by ELISA. All data were shown as mean ± SD by 2-way ANOVA (*n* = 3). Significant difference is indicated by ***^, ###^*p* < 0.001, ****^, ####^*p* < 0.0001(*compared to control group; ^#^compared to COs), and ns (non-significant).

Consistent with previous findings, we observed a more pronounced disintegration of sarcomere structures in HOs following IR injury, in contrast to COs (Fig. 5A). Simultaneously, the intracellular expression of cTnT and cTnI showed a marked reduction in HOs compared to COs (Figs. 5B and S6). Moreover, the release of cTnI, MB, and CKM from HOs began to be released from the organoids 24 h post-IR injury, with the highest levels observed at 72 h and exhibited significantly higher levels than those from COs (Fig. 5C).

In addition, an analysis of inflammatory responses and necrosis-related mRNA levels in both COs and HOs following IR injury revealed a more notable increase in gene expressions within HOs in comparison to COs (Fig. S7A). Furthermore, HOs subjected to IR injury displayed a more significant upregulation of NF-κB, a crucial transcription factor involved in inflammation and processes related to cardiac-vascular damage, in comparison to IR-injured COs (Figs. S7B and S8). The expression levels of phosphorylated ERK, phosphorylated JNK, and phosphorylated p38, which are indicative of increased signaling pathways in cardiac remodeling post-AMI [24, 25], were also significantly elevated in IR-injured HOs compared to COs (Figs. S7C and S8).

### Calcium overload and functional defects in IR-injured HOs

Intracellular calcium overload and subsequent mitochondrial calcium accumulation are observed in acute myocardial ischemia, and these phenomena are exacerbated during reperfusion, ultimately leading to mitochondrial permeability transition pore (mPTP) opening [26]. Furthermore, within a physiological environment, the bulk of calcium during cycles of contraction and relaxation is released from and taken up by the sarcoplasmic reticulum (SR) [27]. Therefore, quantifying SR calcium content is essential in elucidating the pathophysiological mechanisms of calcium overload.

**Fig. 6:**
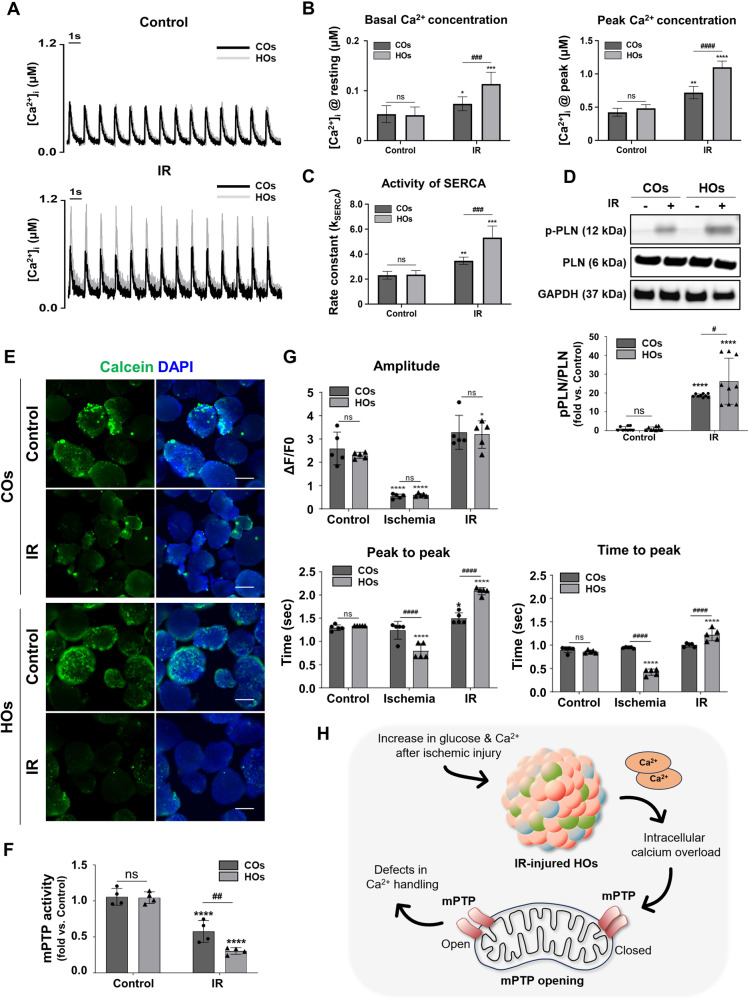
Calcium overload and defects in calcium handling after IR injury. **A** Representative trace of calcium transient in COs and HOs before and after IR injury. control and IR groups. **B** Basal and peak Ca^2+^ concentrations were measured using calcium imaging. All data were shown as mean ± SD by 2-way ANOVA (*n* = 18–20). A significant difference in all graphs are indicated by *^, #^*p* < 0.05, **^, ##^*p* < 0.01^, **^*^, ###^*p* < 0.001, ****^, ####^*p* < 0.0001 (*compared to control group; ^#^compared to COs), and ns (non-significant). **C** The SERCA rate constant, reflecting the activity of SERCA, was calculated by subtracting the reciprocal of the time constant measured after inhibiting SERCA from the reciprocal of the time constant measured in the transient. All data were shown as mean ± SD by 2-way ANOVA (*n* = 18–20). A significant difference in all graphs are indicated by *^, #^*p* < 0.05, **^, ##^*p* < 0.01, ***^, ###^*p* < 0.001, ****^, ####^*p* < 0.0001 (*compared to control group; ^#^com*p*ared to COs), and ns (non-significant). **D** Western blot analysis of phospholamban and phosphorylated phospholamban in COs and HOs before and after IR injury. Quantitative analysis of all western blot data was calculated by the average intensity of the bands in Image J software. Equal protein loading amounts of western blot data were confirmed by GAPDH expression. A significant difference of all graphs is indicated by #,*p < 0.05, ##,**p < 0.01 ###,***p < 0.001, ####,****p < 0.0001(*Compared to control group; #compared to COs), and ns (non-significant). **E** Representative immunofluorescence images of MPTP opening (calcein, green) in control and IR groups. The scale bar represents 200 μm. **F** MPTP opening (calcein) ratio in each group was calculated by image J software. This data was normalized to the control of COs. **G** Beating characteristics of COs and HOs in IR and control groups. Beating analysis was performed by monitoring calcium fluorescence over a period of 20 s under control and IR conditions. A comparison of BPM (beat per minute), peak-to-peak duration, and time-to-peak was performed on COs and HOs in each group. All data were shown as mean ± SD by 2-way ANOVA (*n* = 3). A significant difference in all graphs is indicated by **^, ##^*p* < 0.01, ***^, ###^*p* < 0.001, ****^, ####^*p* < 0.0001 (*compared to the control group; ^#^compared to COs), and ns (non-significant). **H** Schematic summary of findings in (**A**–**F**).

We first observed calcium overload in the organoids by measuring the activity of sarco/endoplasmic reticulum calcium ATPase (SERCA), which significantly influences SR calcium storage [28], to infer the SR calcium content. Before IR injury, there were no significant differences in basal and peak intracellular calcium concentrations between COs and HOs during the contraction–relaxation cycle (Fig. 6A and B). However, after IR injury, both the basal and peak calcium concentrations were significantly higher in HOs compared to COs (Fig. 6A and B). Subsequently, we measured the activity of SERCA (*k*_SERCA_) by calculating the time constant after inhibiting SERCA and observed that the SERCA activity was notably increased in IR-injured HOs compared to IR-injured COs (Fig. 6C). In the same context, the phosphorylated phospholamban (PLN) expression was significantly elevated in IR-injured HOs, confirming the accelerated SERCA activity (Fig. 6D and S9). This suggests that SERCA activity preferentially increases during IR induction in HOs, leading to a significant increase in SR calcium storage in IR-injured HOs compared to IR-injured COs.

To determine whether the IR condition facilitates mPTP opening in HOs, we directly measured fluorescence intensity using an mPTP assay kit in the IR-injured organoids and found a more significant reduction in fluorescence intensity within IR-injured HOs compared to COs (Fig. 6E and F), suggesting that IR injury in HOs leads to a greater increase in mPTP opening than IR injury in COs.

Moreover, the real-time calcium transient assay allowed for an analysis of calcium handling properties in IR-injured organoids (Figs. 6G and S10). Amplitudes showed no significant difference between COs and HOs during ischemia and IR injury (Fig. 6G). However, HOs subjected to ischemia-injury exhibited aberrant beating properties (Fig. 6G) with a significant change in beating rate (peak-to-peak) and systolic time (time-to-peak), indicated a disease-like model. Consistent with the ischemia results, the IR-injury condition induced further detrimental calcium handling properties in HOs, resulting in a decrease in beating and systolic parameters compared to those of COs (Fig. 6G). Collectively, these results indicate that multicellular HOs effectively mimic clinically observed AMI pathologies, such as calcium overload and mPTP opening under IR conditions, as well as the mimicking defects in calcium handling function (Fig. 6H).

### Modeling cardiac fibrosis in IR-injured HOs

**Fig. 7:**
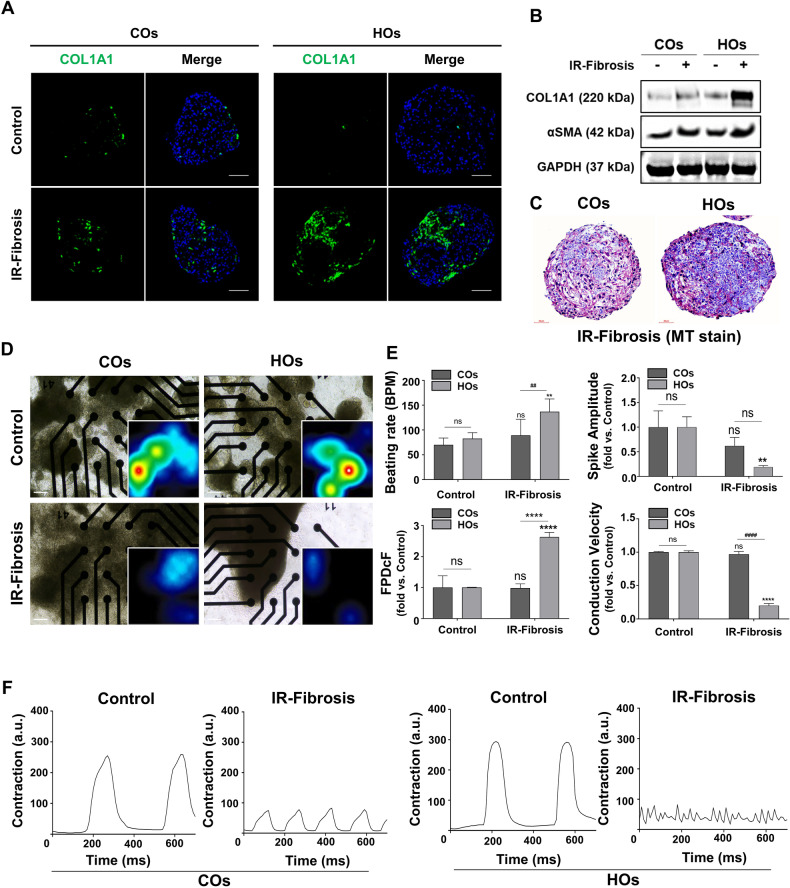
Fibrosis induction and functional defects in AMI-organoids. **A** Representative immunofluorescence images of COL1A1 (green) and DAPI (blue) in each group. The scale bar represents 100 μm. **B** The protein expression of COL1A1 and α-SMA, which are fibroblast activation and fibrosis indicators using western blot in cell lysates from COs and HOs in each group. Equal protein loading amounts were confirmed by GAPDH expression. **C** The morphologies of the IR-Fibrosis organoid by Masson’s Trichrome staining. The scale bar represents 40 μm. **D** Evaluation of the electrophysiological function of COs and HOs on the electrode of the MEA plate in each group. Magnified image to show a heatmap of a representative MEA recording. The spike activity of each active electrode is color-coded: white/red represents high spike activity; blue/black represents low spike activity. **E** Beating rate (BPM), Spike amplitude, FPDcF, and conduction velocity of COs and HOs in each group through MEA recording. All data were shown as fold-change, as mean ± SD, by 2-way ANOVA (*n* = 3). A significant difference of all graphs is indicated by ^#,^*p < 0.05, ^##,^**p < 0.01, ^###,^***p < 0.001, ^####,***^*p < 0.0001(*Compared to control group; ^#^compared to COs) and ns (non-significant). **F** Comparison of contraction in control COs versus IR-fibrosis COs and control HOs versus IR-fibrosis HOs.

Cardiac fibrosis is a consequential outcome of cardiac remodeling following AMI [29]. To replicate the cardiac fibrosis within IR-injured HOs, we cultured the organoids with 10 μM TGF-β1 for 7 days. Staining for COL1A1, a marker indicating fibrosis progression, revealed a more pronounced accumulation of collagen in IR-injured HOs after fibrosis induction compared to IR-injured COs (Fig. 7A and S12A). This substantial collagen accumulation in IR-injured HOs was further confirmed through western blot analysis and Masson’s trichrome (MT) staining (Fig. 7B, C and S12B). Additionally, we assessed increased expression levels of mRNA associated with fibrosis-related genes (ACTA2, POSTN, Vimentin, MMP2) and collagen-related genes (PAI1, COL1A1, COL1A2, COL3A1) in IR-injured HOs after fibrosis induction using quantitative PCR (Fig. S12C).

To validate the alterations in calcium handling in HOs under the IR-fibrosis condition, we conducted a calcium transient assay. Real-time video recordings allowed for an analysis of beating properties (Fig. S13A and Videos 11, 12) of the organoids, and HOs subjected to IR-fibrosis displayed an increase in amplitude and beating (peak-to-peak) but a decrease in systolic (time-to-peak) compared to IR-fibrosis COs (Fig. S13B), reflecting a form of arrhythmic event in the human heart.

Heart disease also leads to alterations in electrophysiological properties [30, 31]. To demonstrate the defect of electrophysiological characteristics in IR-fibrosis HOs, multielectrode arrays (MEA) were utilized (Fig. 7D). Consistent with the analysis of beating properties in calcium transient, the beating rate (BPM) was significantly increased in IR-fibrosis HOs compared to IR-fibrosis COs, and the BPM exceeded 100, a characteristic of tachycardia (Fig. 7E). The spike amplitude, indicative of action potential height, demonstrated a decline in both COs and HOs within the IR-fibrosis group, but HOs displayed a particularly significant difference compared to COs (Fig. 7E). Measurement of the field potential duration corrected by Fridericia’s formula (FPDcF) unveiled a twofold increase in HOs subjected to IR-fibrosis (Fig. 7E), mirroring the prolongation of the period between the onset of the Q wave and the conclusion of the T wave. The cardiac conduction velocity exhibited a significant slowing in IR-fibrosis HOs compared to IR-fibrosis COs, resulting from an elevated risk of re-entrant excitation attributing to collagen accumulation in HOs (Fig. 7E). Additionally, induction of fibrosis subsequent to IR injury resulted in reduced contractility of COs but maintained normal cardiac rhythms, whereas, in HOs, it led to both diminished contractility and irregular cardiac rhythms (Fig. 7F).

### Further confirmation of heart disease modeling in HOs

**Fig. 8:**
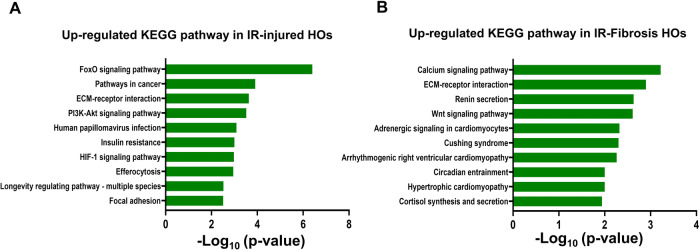
Comparison of up-regulated KEGG pathway of control HOs versus IR-injured HOs and control HOs versus IR-fibrosis HOs. **A** Visualized graphs of up-regulated KEGG pathways in IR-injured HOs compared to control HOs. **B** Visualized graphs of up-regulated KEGG pathways in IR-fibrosis HOs compared to control HOs. The pathways were selected based on criteria including a fold change >2 and a *p*-value less than 0.05.

The QuantSeq 3’ mRNA-Sequencing analysis provided further support for the modeling of AMI and cardiac fibrosis in HOs (Fig. 8). In comparing the up-regulated KEGG pathways between control HOs and IR-injured HOs (Fig. 8A), it was observed that the FoxO signaling pathway, known to be activated in cardiomyocytes under ischemic stress [32], was predominantly up-regulated. Additionally, pathways associated with cancer, which share common systemic pathology and mechanisms with heart failure [33], were also up-regulated in IR-injured HOs. Furthermore, pathways related to extracellular matrix (ECM)-receptor interaction, PI3K-Akt signaling, and HIF-1 signaling were up-regulated in IR-injured HOs compared to control HOs which are known to play crucial roles in cardiac remodeling [34], alleviating negative post-infarct changes in myocardium [35], and modulating post-infarct healing after myocardial ischemic injury [36], respectively. Interestingly, pathways associated with human papillomavirus, insulin resistance, efferocytosis, longevity genes, and focal adhesion kinase (FAK) inhibition were also found to be up-regulated in IR-injured HOs. These pathways are implicated in various processes such as the diagnosis of myocardial infarction [37], hypoxia-induced inhibition of angiogenesis [38], macrophage-mediated clearance of dead cells during myocardial infarction [39], modulation of cardiovascular function [40], and regulation of cardiac fibrosis post-MI [41].

The comparison between control HOs and IR-fibrosis HOs revealed up-regulated pathways associated with cardiac fibrosis (Fig. 8B). One of the pathways identified as up-regulated in IR-fibrosis HOs, is the calcium signaling pathway, known to play a role in fibroblast activation by increasing intracellular calcium concentration, promoting fibroblast proliferation and migration, and inducing the synthesis of extracellular matrix proteins [42]. Extracellular matrix (ECM) remodeling is closely linked to cardiac remodeling and the development of heart failure [43]. Furthermore, the renin-angiotensin-aldosterone system (RAAS) pathway, Wnt signaling pathway, and adrenergic signaling pathway were also identified as up-regulated in IR-fibrosis HOs. These pathways are known to promote myocardial fibrosis and cardiac remodeling, contributing to the progression of heart failure [44–47], respectively. Additionally, conditions such as Cushing syndrome, circadian disruption, and cardiomyopathy, which are associated with increased myocardial fibrosis [48–50], were found to be relevant to the up-regulated pathways in IR-fibrosis HOs. Cortisol is also experimentally shown to induce cardiomyocyte hypertrophy [51].

## Discussion

This study demonstrates the successful generation of multi-cellular heart organoids (HOs) from hiPSCs through in vitro self-organization. In native development, structural maturation is orchestrated by cell-cell interactions and associated physical transformations [52]. Crucially, these interactions initiate during early development [53]. Reports indicate that factors secreted by other cells, such as endothelial cells, fibroblasts, and inflammatory cells, facilitate the maturation of functional and structural cardiomyocytes [54]. Consequently, we devised HOs, leveraging differentiation signals within 3D embryoid bodies to prompt multicellular configuration, thereby achieving optimal adult-like maturation timelines.

Traditionally, disease modeling using cell models has primarily centered around genetic disorders, while attempts to recreate adult-onset conditions in vitro have been limited. Notably, a recent experiment involving oxygen gradients generated organoids with infarct-like characteristics, showing various disease features [17]. However, these methods do not authentically replicate the intricate pathology of myocardial infarction (MI) because they tend to focus on replicating specific events, such as ischemia. Conversely, our study took a systematic approach by inducing ischemia and ischemia-reperfusion (IR) injury in HOs through precisely defined chemical culture conditions.

Hypoxia chambers have traditionally been utilized for inducing hypoxia in in vitro cell cultures. However, a challenge arises from the fact that hypoxia-inducible factors, which act as sensors of hypoxia and regulators of cellular hypoxic responses, are continuously synthesized under hypoxic conditions but rapidly degraded upon returning the cell culture to normoxia conditions. To address this challenge, we employed a widely used hypoxia-inducing agent, cobalt chloride (CoCl_2_), to induce hypoxia in HOs. CoCl_2_ effectively stabilized the expression of hypoxia-inducible factors 1α and 2α during prolonged culture of HOs. This approach ensures a continuous induction of hypoxia, providing a more sustained environment compared to traditional hypoxia chambers. Additionally, by subsequent rapid nutrient supply and calcium overload in the CoCl_2_-treated HOs, we effectively induced a response in HOs that closely resembled the physiological changes observed in human MI. These IR-induced HOs exhibited characteristic features of acute AMI, including specific cell death in the myocyte population, release of relevant biomarkers, increased inflammatory responses, and functional impairments.

We suggest that HOs may exhibit a more pronounced apoptotic response to hypoxic conditions compared to COs. Ischemia-reperfusion (IR) injury, which is a primary driver of cardiac disease, is known to primarily induce apoptosis-induced cell death, particularly affecting endothelial cells more than cardiomyocytes [55]. Endothelial cells also play a crucial role in modulating the inflammatory responses associated with IR injury and myocardial infarction [56] Thus, we presume that the increased population of endothelial cells in HOs may contribute to the more sensitive responses to hypoxia-induced apoptosis and the heightened production of inflammatory factors observed after IR injury. This highlights the advantage of utilizing HOs for modeling cardiac diseases and studying their pathophysiological mechanisms, particularly in the context of ischemic conditions.

Certainly, the success in mimicking acute MI and cardiac fibrosis using HOs in this study is a significant step forward. However, considerations should be given to incorporating additional cell types, such as mural cells (pericytes and vascular smooth muscle cells) and immune cells (myeloid and lymphoid cells), as their interactions play a role in human cardiovascular diseases [56, 57]. Nonetheless, the HOs in this study demonstrated the successful generation of self-organized major cellular compositions found in the ventricles of human hearts. Notably, heart diseases such as acute AMI and cardiac fibrosis were effectively recapitulated under defined culture conditions, showing defects in phenotypes and functional impairments. While many studies have reported heart disease induction at the cellular level, this study stands out by uniquely demonstrating the impairment of calcium ion handling, electrical activity, and contractility. These specific observations provide a comprehensive reflection of cardiac functions. As a result, this study introduces a promising heart organoid model derived from hiPSCs.

### Supplementary information

Supplementary methods, figures, table and references

Original Data File

Supplement video 1

Supplement video 2

Supplement video 3

Supplement video 4

Supplement video 5

Supplement video 6

Supplement video 7

Supplement video 8

Supplement video 9

Supplement video 10

Supplement video 11

Supplement video 12

## Data Availability

The datasets generated during and/or analyzed during the current study are available from the corresponding author on reasonable request.
