# Supplementary material for: Modeling acute myocardial infarction and cardiac fibrosis using human induced pluripotent stem cell-derived multi-cellular heart organoids
Source: Cell Death Dis. 2024 May 1;15(5):308. doi: 10.1038/s41419-024-06703-9 (PMC11063052; doi:10.1038/s41419-024-06703-9)
Supplement: Supplementary file 1 — Supplementary methods, figures, table and references [file 41419_2024_6703_MOESM1_ESM.pdf]

Supplementary Information

Supplementary materials & methods.

Expansion and maintenance of human induced pluripotent stem cells (hiPSCs)

The same hiPSCs in our previous study (1) were utilized for this research. These established hiPSCs were expanded and cultured under feeder-free conditions on Matrigel (Corning, USA) coated T75 tissue culture flasks in the presence of mTeSR1 (Stem Cell Technologies, Canada). The culture medium was changed every day. Once the hiPSCs culture was reached at 70-80% confluence, they were passaged using TrypLE dissociation buffer (Invitrogen, USA). The cells were cultivated at a temperature of 37°C within a 5% CO2 incubator.

Generation of cardiac organoids (COs) and heart organoids (HOs)

To differentiate hiPSCs into 3D cardiac and heart organoids, we employed the embryoid body (EB) formation method and cultivated them in ultra-low attachment (ULA) 6-well plates at a density of approximately $2.0x10^6$ cells per well. The seeded hiPSCs were cultured for 4 days in mTeSR1 medium supplemented with 10μM Y27632 (ROCK inhibitor), facilitating the formation of 3D aggregates.

Cardiac organoid (CO) differentiation was initiated approximately 4 days after seeding (D0) and performed through small-molecule control of the Wnt/β-catenin pathway with 6 μM GSK3 inhibitor in RPMI 1640 supplemented with B-27 without insulin for 48 hours and inhibition of Wnt/β-catenin pathway for 48 hours. On day 8, the medium was changed to RPMI 1640 medium with B-27 supplementation without vitamin A by changing the medium every 2 days. The COs were maintained in RPMI 1640 medium with B-27 supplementation without vitamin A until day 30 (D30) as described in our previous report (1).

To differentiate heart organoids (HOs), we initially applied the differentiation procedures used for COs to induce mesodermal differentiation from EBs. However, the differentiated mesoderm was subsequently cultured with 30 ng/ml of BMP, 30 ng/ml of VEGF, 30 ng/ml of FGF, and 10 μM TGF-beta/Smad inhibitor-containing medium from D8 to D14 to induce differentiation into cardiomyocytes, endothelial cells, and fibroblasts. Following multi-lineage induction, the HOs were maintained in RPMI 1640 medium supplemented with 10 ng/ml of TGFβ, and B-27 without vitamin A until day 30 (D30).

The beating efficiency of differentiating COs and HOs during the culture periods was assessed by analyzing videos obtained at 40× magnification using an ECLIPSE Ts2 microscope (Nikon, Tokyo, Japan). Videos capturing continuous beating of COs and HOs were recorded for 14–15 seconds on

days 8, 10, 12, 15, 20, and 30 of differentiation. The number of beating organoids was then quantified from the recorded videos.

Flow cytometry analysis

For flow cytometry analysis, the 3D COs and HOs were dissociated into single cells using 0.05% trypsin-EDTA for 15 minutes at 37℃ in incubators. The dissociated single cells were then passed through a 100-μm Nylon cell strainer (Corning, USA). To prepare the cells for fixation and permeabilization, they were treated with Fix/Perm Solution from BD Biosciences (USA) or 4% paraformaldehyde for 20 minutes at 4℃. Subsequently, the cells underwent three washes with 1x wash buffer from BD Bioscience or 2% FBS in DPBS.

After the washes, the cells were incubated overnight at 4℃ with primary antibodies (cTnT, VE-CAD, CD90, CD31) in 1x wash buffer or 2% FBS in PBS. Following another three washes with 1x wash buffer or 2% FBS in DPBS, the cells were exposed to Alexa Fluor 488 secondary antibodies for 1 hour at room temperature. Following this incubation, the cells were subjected to additional three washes with 1x wash buffer or 2% FBS in DPBS. The resulting cell suspension was then analyzed using the ACEA NovoCyte-3000 Flow cytometer (ACEA Bioscience, USA). Specific dilution ratios and antibody information for this study are provided in Supplementary Table 1.

Single cell RNA-sequencing (scRNA-seq)

For the scRNA-seq analysis, the 3D COs and HOs collected on day 24 were dissociated into single cells using the gentlyMACS Dissociator and EBDK enzyme from Miltenyi Biotec (Germany). Subsequently, scRNA-seq was performed by Ebiogen (Korea) to compare the gene expression profiles of COs and HOs, and the libraries were prepared using the 10X Genomics Next GEM technology. The Winseurat program was employed for clustering and analyzing the data sets of hiCOs and hiHOs.

In the scRNA-seq analysis, a total of 3555 cells form COs and 2967 cells from HOs were counted. UMAP clustering was carried out on the cells after applying Cell Filtering to eliminate low-quality cells. UMAP clustering involved 2968 cells from COs and 2587 cells from HOs for single-sample clustering, as well as 2970 cells from COs and 2588 cells from HOs for differential expression analysis between samples. In the UMAP analysis, data sets with over 2000 transcripts per cell were initially filtered, and clustering was performed using 10 principal components (PCs) and a resolution of 0.8.

The single-sample clustering resulted in 14 clusters for COs and 12 clusters for HOs. To identify cells corresponding to each cluster, representative markers of cardiac constituent cells (CM, CF, EC) were examined. For further specification of cell types, violin plots displaying significant gene differences were compared, and the top 20 genes expressed in each cluster were referred to from the pangiaoDB database.

In the integration clustering of the two samples for differential expression analysis between samples, a total of 15 clusters were obtained. Similar to before, representative markers of cardiac constituent cells were examined to identify cells corresponding to each cluster. The differential expression analysis was performed between the two samples in each cell type using the ExDEGA (Excel-based DEG Analysis) analysis tool provided by Ebiogen.

Quantitative real-time polymerase chain reaction

On day 24 of differentiation, total RNA was extracted from the COs and HOs using the traditional TRIzol reagent method (Thermo Fisher Scientific, USA). Prior to the TRIzol extraction, the COs and HOs underwent ultrasonication for 4 cycles on ice using a sonicator (Sonoplus mini-20; wave output power: 320W, 20 seconds on/off, ultrasonic on/off cycle: 0.5 minutes). Quantification of the total RNA was performed using a SpectraMax iD5 Microplate Reader (Molecular Devices, USA). Subsequently, total cDNA was synthesized through reverse-transcription polymerase chain reaction (PCR) using a CellScript All-in-One 5X First Strand cDNA synthesis Master Mix (Cellsafe, Korea) according to the manufacturer's instructions.

For real-time quantitative PCR (qPCR), the synthesized cDNA was amplified and quantified using the TB Green Premix Ex Taq (Tli RNaseH Plus) Kit (Takara, Japan) on the CFX Connect Real-Time PCR detection system (BioRad, USA). The amplification reactions followed this protocol: 95°C for 30 seconds, 44 cycles at 95°C for 10 seconds, 60°C for 30 seconds, dissociation at 55°C to 60°C for 5 seconds based on each primer, and 95°C for 5 seconds. To normalize relative quantification in each expression level, GAPDH was utilized as a housekeeping gene for intracellular reaction control. The quantification was calculated using the $2^{-\Delta\Delta CT}$ protocol.

The genes of interest encompassed those related to hypoxia, cardiac structure molecules, inflammation, apoptosis, necrosis, myofibroblasts, collagen-related genes, hypertrophy, and cellular metabolism during AMI. The primer sequences used for qPCR in this study are specified in Supplementary Table 2.

Immunostaining

The organoids were fixed by incubating them overnight at 4℃ with 4% paraformaldehyde in PBS. Following fixation, the organoids were stored in a 20% sucrose solution at 4℃ until they sank in the cornical tube. Once the organoids had sunk, they were embedded in OCT compound (Sakura Finetek, USA) to prepare frozen sections. The blocks of OCT were sliced using a microtome at 10 μm thickness. These sectioned slides were then stored at -80℃ in a deep freezer until analysis.

To analysis the dissociated cells from organoids, The COs and HOs were dissociated into single cells using gentleMACS™ Tissue Dissociators (Miltenyi, Germany). This process involved using 0.05%

trypsin-EDTA and the device's program to desorb the cells into single-cell form. The single cells were then seeded onto a culture plate, and after one week, immunofluorescence staining was conducted.

In the staining process, the organoid cryosections or cultured cells underwent washing with PBS and blocking in 10% normal donkey serum for 1 hour at room temperature. Primary antibodies were incubated with the samples overnight at 4℃. These primary antibodies were diluted in 1% normal donkey serum. After washing the samples with PBS, secondary antibodies were applied in PBS for 1 hour and 30 minutes. The primary antibodies used included cTnT (abcam, ab8295, 1:100), α-actinin (abcam, ab9465, 1:100), VE-cad (R&D system, AF938, 1:50), cleaved caspase3 (Cell Signaling, 9661, 1:200), COL1A1 (Cell Signaling, 39952, 1:400), Vimentin (abcam, ab137321, 1:200), MLC-2a (synaptic systems, 311-011, 1:500), and MLC-2v (abcam, ab79935, 1:500). The acquired images were visualized and processed using a Nikon laser scanning fluorescence microscope (Nikon, Japan), with further analysis performed using ImageJ.

For the comprehensive 3D analysis, the organoids were made transparent using the Tissue Clearing™ kit (Binaree, Korea). The incubation time was extended, and additional antibodies were applied following the same method as the previous approach. Representative images of the transparentized organoids were captured using the LSM980 imaging system (Carl Zeiss, Germany).

TUNEL assay

To assess cell apoptosis, an in situ cell death detection kit (Roche, Switzerland) was employed. For this purpose, frozen sections were subjected to an incubation with 0.5% Triton-X100 at room temperature for a duration of 15 minutes. Subsequently, the reagents from the TUNEL detection kit were combined and then applied to the samples, which were incubated at 37℃ for a period of 1 hour. Finally, images of the samples were visualized and processed using a Nikon laser scanning fluorescence microscope (Nikon, Tokyo, Japan), with further analysis carried out using the ImageJ software.

Masson’s trichrome staining

To visually confirm the fibrosis induction in organoids, they were fixed using 4% paraformaldehyde and stained with eosin. Subsequently, the stained organoids were embedded in Histogel, and these Histogel blocks were further processed using a standard paraffin embedding procedure. The resulting paraffin blocks were then sectioned using a microtome. For the paraffin slides, the paraffin was removed using xylene. The paraffin-removed slides were subjected to Masson’s Trichrome (MT) staining in order to assess fibrosis. Images of the stained slides were captured using a slide scanner (Motic, USA) and processed with the Motic DSAssistant program.

Western blot analysis

On day 24 of differentiation, organoids were collected in radioimmunoprecipitation assay (RIPA) lysis buffer containing a protease and phosphatase inhibitor cocktail. These collected samples were then ultrasonicated for 4 minutes on ice using a sonicator (Sonoplus mini-20; 20 s on/off, ultrasonic on/off cycle: 0.5 minutes, wave output power: 320 W). The protein content of the samples was quantified using a bicinchoninic acid protein assay kit (Thermo Fisher Scientific, USA) to ensure equal protein concentrations for loading. Subsequently, 20 μg of protein from COs and HOs was separated on a gradient of 4% to 12% Tris-glycine gels (Thermo Fisher Scientific) and electrophoretically transferred to 0.2 μM nitrocellulose transfer membranes using an iBlot 2 Dry Blotting System (Thermo Fisher Scientific). The membranes were blocked with 5% skim milk and then probed with appropriate primary antibodies (1:500 to 1:1,000) for 30 minutes to overnight at 4℃. Afterward, the membranes were incubated with polyclonal anti-rabbit/mouse horseradish peroxidase-conjugated secondary antibodies for 1 hour at room temperature. Finally, the membranes were developed using a Pierce ECL Western Blotting substrate.

ELISA for analyzing AMI-related biomarkers

Organoid culture supernatants were subjected to analysis using ELISA kits for cardiac troponin I (cTnI) from Ray Biotech (ELH-CTNI), creatine kinase-MB (CKM) from Abcam (ab264617), and myoglobin (MB) from Abcam (ab171580), following the manufacturer's instructions. Each kit includes a plate that has been coated with an anti-human target antibody. Standards and samples were incubated on the coated plate, followed by the addition of secondary antibodies and horseradish peroxidase (HRP). The absorbances of the samples were measured using a plate reader at a wavelength of 450nm. This assay provides information about the levels of these specific cardiac biomarkers in the cell supernatants.

Intracellular calcium measurements using calcium imaging

Intracellular calcium concentration of organoids was measured using PTI system (PTI, Lawrenceville, NJ). Organoids were incubated with 2 μM fura-2-acetoxymethyl ester (fura-2-AM) for 30 min at room temperature. After sedimentation, the supernatant was removed, and organoids were reintroduced into a perfusion solution containing 500 μM Ca^{2+} for 10 min. Then, the fura-2-AM-loaded organoids were placed in a bath with field stimulator and excited by 340 and 380 nm filtered fluorescent light. The emitted signal was measured simultaneously with photomultiplier tubes. *In situ* calibration was conducted to convert the fluorescence signals measured at two excitation wavelengths into calcium concentrations. After each experiment, cells were permeabilized under identical conditions and then exposed to 0 Ca solution (141.4 mM NaCl, 4 mM KCl, 0.1 mM EGTA, 5 mM $MgCl_2$, 5.5 mM glucose, and 10 HEPES with pH 7.4) and 5 La solution (141.4 mM NaCl, 4 mM KCl, 0.1 mM EGTA, 5 mM $MgCl_2$, 5.5 mM glucose, and 10 HEPES with pH 7.4) to measure the intensity of fluorescence and its ratio.

Based on these measurements, the intracellular calcium concentration was calculated using the following equation:

$$[Ca^{2+}]_i = K_d\left(\frac{R - R_{min}}{R_{max} - R}\right)\left(\frac{F_{380,max}}{F_{380,min}}\right)$$

The K_d value represents the dissociation constant of the binding between fura-2 and calcium, $F_{380,max}$ and $F_{380,min}$ represent the maximum and minimum fluorescence intensities at a wavelength of 380 nm, respectively, R_{min} is the fluorescence intensity ratio in the 0 Ca solution, and R_{max} is the product of the fluorescence intensity ratio in the 5 La solution and a converting factor. Measurements from at least 20 steady-states calcium transients were averaged for each organoid for each stage of the experimental protocol. All experiments were performed in a temperature-controlled bath solution at 37 ± 1°C.

Measuring SERCA activity

Due to the characteristic difficulty of solution diffusion in organoids compared to single cardiac cells, we were unable to observe synchronized calcium release using fast caffeine treatment. Therefore, we employed an alternative method to quantify sarcoplasmic reticulum (SR) calcium overload. We defined the time constant of decay of intracellular calcium concentration ($[Ca^{2+}]_i$) as τ_{tran}, measured when field stimulation was applied. This time constant reflects the activity of intracellular calcium removal mechanisms in the organoid, including SERCA (Sarco/Endoplasmic Reticulum Ca^{2+}-ATPase) and NCX (Na^+/Ca^{2+} exchanger). After stabilization, cells were treated with 2.5 μM thapsigargin for more than 10 minutes to inhibit most of the SERCA activity completely, and then the time constant was measured again (τ_{thap}), reflecting the activity of calcium removal mechanisms excluding SERCA. To quantify the relative contribution of Ca^{2+} removal, the reciprocal of the time constant was taken and converted into rate constants (k_{tran}, k_{thap}). The difference between these two rate constants was defined as the activity level of SERCA (k_{SERCA}).

Mitochondrial permeability transition pore (mPTP) assay

The mPTP assay was performed using the Image-IT™ LIVE Mitochondrial Transition Pore Assay Kit (Invitrogen, USA), following the manufacturer's instructions. Briefly, a labeling solution containing 1mM calcein AM, 1mM Hoechst 33342, and 1M $CoCl_2$ was applied to the organoids and incubated at 37℃ for 30 minutes. Representative images were captured using a Nikon laser scanning fluorescence microscope and subsequently processed using the NIS Element program.

Calcium transient assay

To detect intracellular calcium influx in organoids, FLIPR Calcium 6 dye from Molecular Devices (USA) was utilized. On the day of the assay, the organoids were gently suspended in 200 μL of culture medium and transferred to a 48-well plate. To load the calcium dye, FLIPR Calcium 6 dye was mixed at a 1:20 dilution in culture medium. After removing 20 μL of the culture medium from each well, 20 μL of the diluted FLIPR Calcium 6 dye was added. The 48-well plate was then incubated at 37°C for 30 minutes prior to detecting intracellular calcium influx. Images capturing calcium influx were obtained using a Nikon laser scanning fluorescence microscope equipped with a 10X objective. Representative images were acquired for 20 seconds, and quantitative analysis of the images was performed using NIS-Elements AR software version 5.3 from Nikon. This assay allowed for the visualization and measurement of intracellular calcium changes in the organoids.

Multielectrode array (MEA) analysis

To analyze the electrophysiological functions of organoids, multielectrode array was performed. The microelectrode plate was coated with a solution of 100 μg/ml fibronectin, 37℃ for 1 hour. After removing the coating solution, an appropriate number of organoids were suspended in RPMI 1640 medium (Thermo Fisher Scientific, USA) and seeded onto the coated plate. The seeded organoids were then cultured for an additional 3 to 4 days. The results of the assay were acquired using the Maestro MEA system (Axion Biosystems, USA) and analyzed using the Axis software.

Analysis of contraction using MUSCLEMOTION

MUSCLEMOTION quantifies contraction in arbitrary units based on pixel intensity changes. For MUSCLEMOTION analysis, we installed a muscle motion software in image J, referring to the paper by Sala, L et al (2). MUSCLEMOTION source code was obtained from https://github.com/l-sala/MUSCLEMOTION/blob/master/MUSCLEMOTION%20v1.0.ijm. To accurately measure organoid contraction, movies captured images at 100 frames per second using a microscope (Nikon, Japan) at 20x magnifications. The movies were modified by referring to the inter-frame distance, algorithm description, and advanced options and analyzed in image J with MUSCLEMOTION source code.

Library preparation and sequencing of QuantSeq 3' mRNA-Seq

For Quant 3' mRNA-seq, RNA isolation was performed as previously described for quantitative RT-PCR. For control and test RNAs, the construction of library was performed using QuantSeq 3' mRNA-Seq Library Prep Kit (Lexogen, Inc., Austria) according to the manufacturer's instructions. In brief, each total RNA were prepared and an oligo-dT primer containing an Illumina-compatible sequence at its 5' end was hybridized to the RNA and reverse transcription was performed. After degradation of the RNA template, second strand synthesis was initiated by a random primer containing an Illumina compatible

linker sequence at its 5’ end. The double-stranded library was purified by using magnetic beads to remove all reaction components. The library was amplified to add the complete adapter sequences required for cluster generation. The finished library is purified from PCR components. High-throughput sequencing was performed as single-end 75 sequencing using NextSeq 550 (Illumina, Inc., USA).

Data analysis of QuantSeq 3’ mRNA-Seq

QuantSeq 3’ mRNA-Seq reads were aligned using STAR (3). STAR indices were either generated from genome assembly sequence of the representative transcript sequences for aligning to the genome and transcriptome. The alignment file was used for assembling transcripts, estimating their abundances, and detecting differential expression of genes. Differentially expressed gene were determined based on counts from unique and multiple alignments using coverage in HTSeq-count (4). The RC (Read Count) data were processed based on TMM+CPM normalization using EdgeR methods. Gene classification was based on searches done by DAVID (http://david.abcc.ncifcrf.gov/) and Medline databases (http://www.ncbi.nlm.nih.gov/). The KEGG (Kyoto Encyclopedia of Genes and Genomes) pathway analysis of DEGs (Differential Expression Genes) was performed using ExDEGA (Ebiogen, Inc., Korea) and DAVID. DEGs analysis was performed by selecting significant genes that met the conditions of fold-change > 2, p-value < 0.05, and normalized data > 4. graphic visualization was performed using Prism 10 (Dotmatics, Inc., USA) and showed the top 10 KEGG pathways.

Supplementary Figures

Supplementary figure 1. Tracking cardiomyocyte subtypes during organoid differentiation periods. Representative immunostaining images for MLC-2a and MLC-2v of differentiating COs and HOs from D5 to D24. The scale bar represents 100 μm.

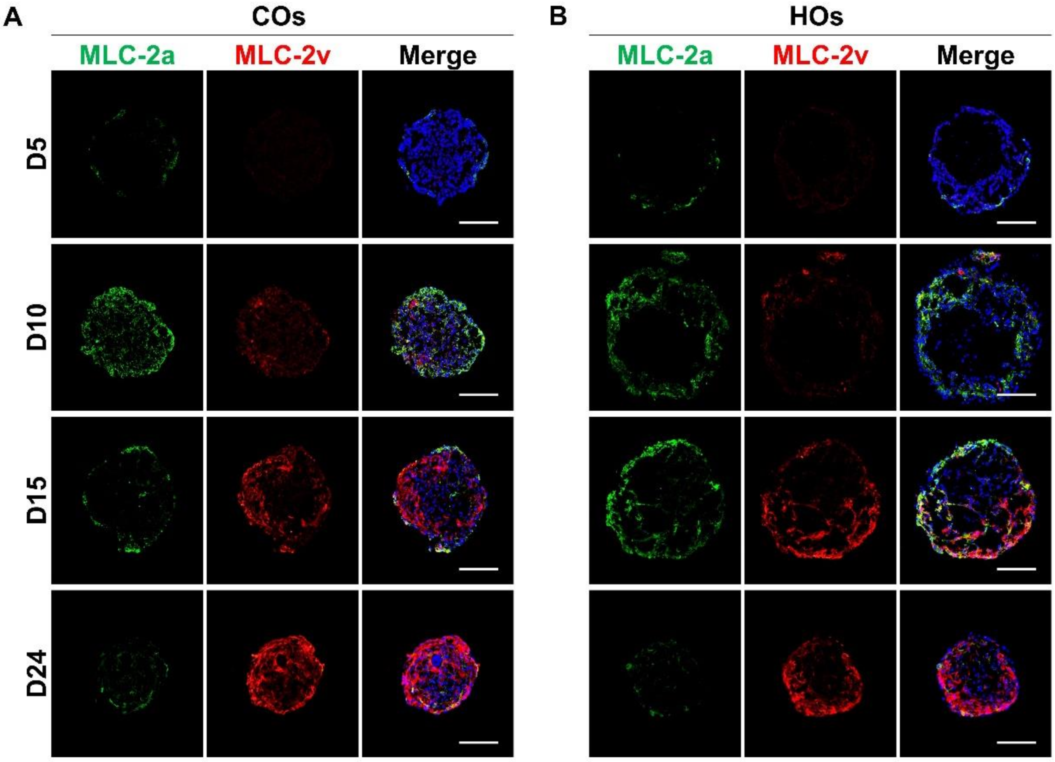

Supplementary figure 2. FACS analysis of COs and HOs. Representative result of FACS analysis for cardiomyocyte marker (cTnT), fibroblast marker (CD90), and endothelial cell marker (VE-Cad) in COs and HOs on Day 30.

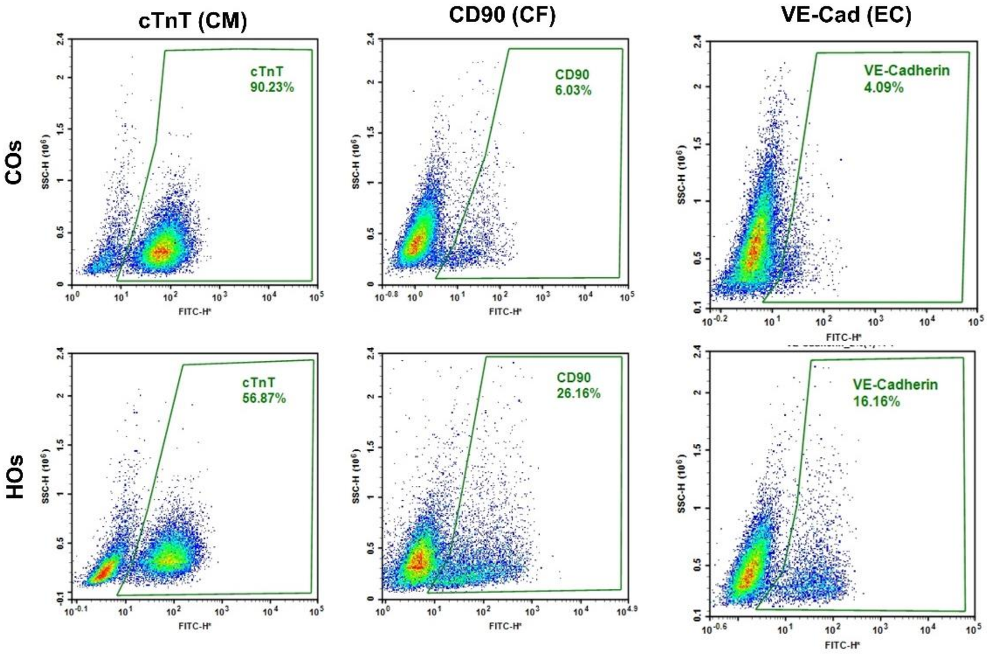

Supplementary figure 3. Original western blot images of Figure 4B. Bands in blue boxes were incorporated in this study. Another experimental condition was not described in this study.

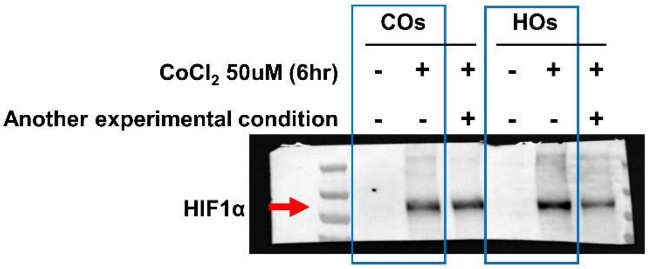

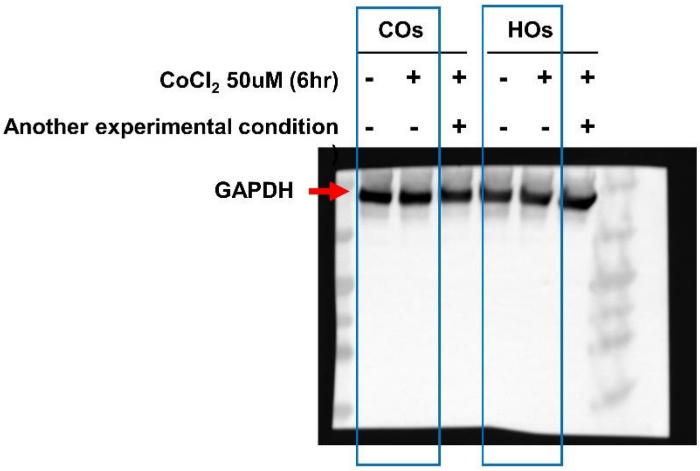

Supplementary figure 4. Original western blot images of Figure 4D.

Cleaved casplase-3

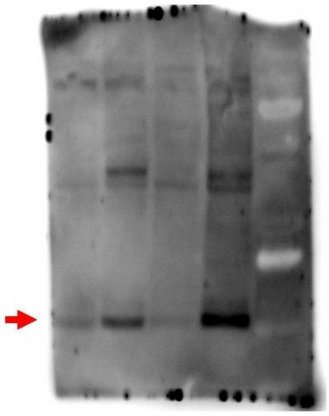

Caspase-3

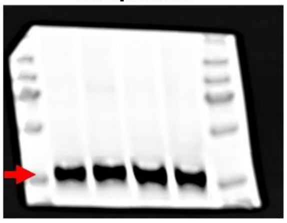

GAPDH

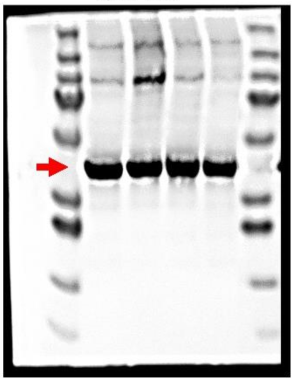

Supplementary figure 5. mitochondrial apoptosis after IR injury. (A) Western blot analysis in cell lysates from COs and HOs in control and IR groups. The protein expression of Bax and Bcl2, which are mitochondrial death was calculated by the average intensity of the bands in Image J software. Data normalized to that of Bcl2. Equal protein loading amounts were confirmed by GAPDH expression. A significant difference is indicated by $^{\#\#\#\#,****}p < 0.0001$ (*Compared to control group; #compared to COs) and ns (non-significant). (B) Original western blot images of (A). Bands in blue boxes were incorporated in this study. Another experimental conditions was not described in this study.

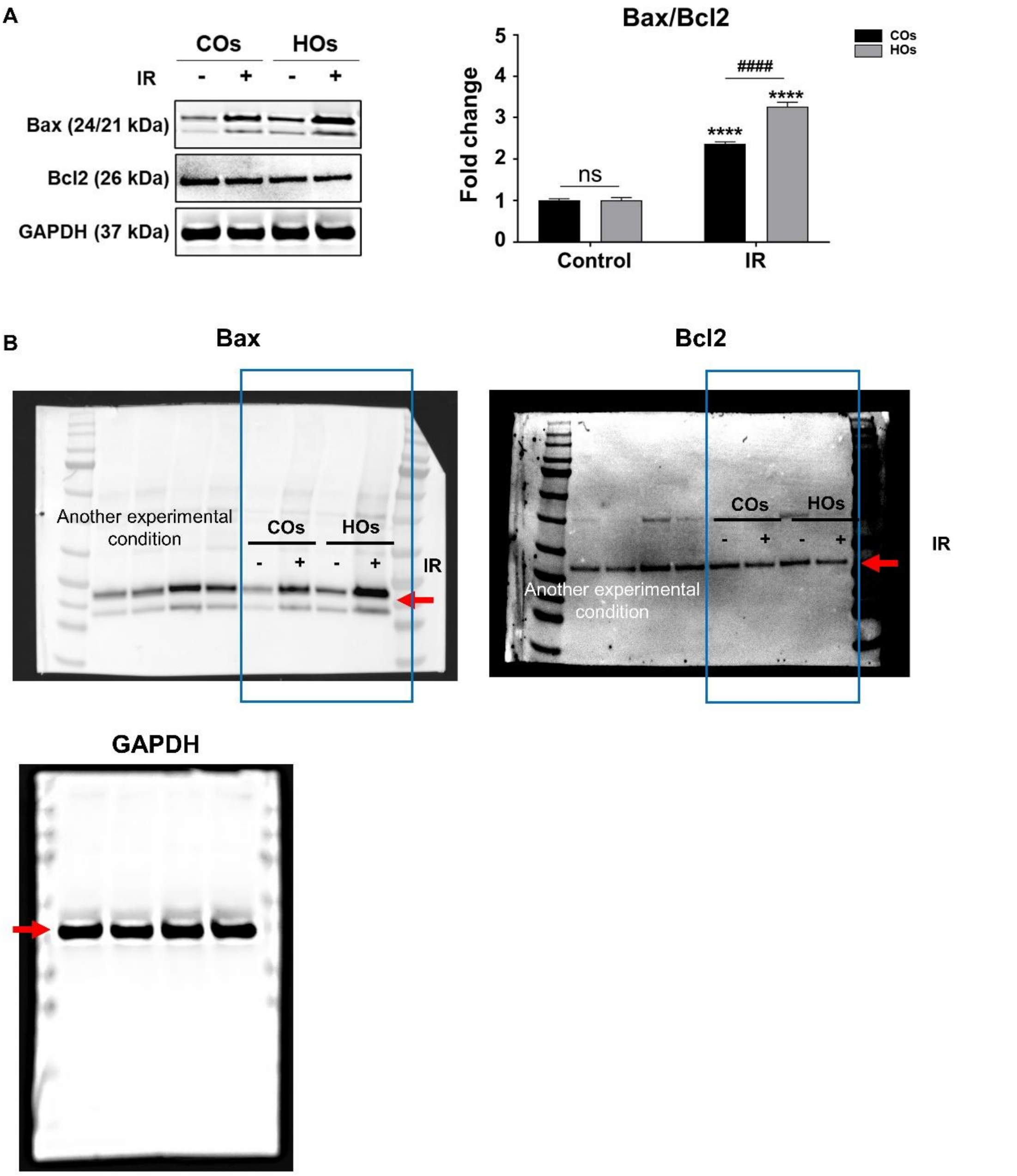

Supplementary figure 6. Original western blot images of Figure 5B.

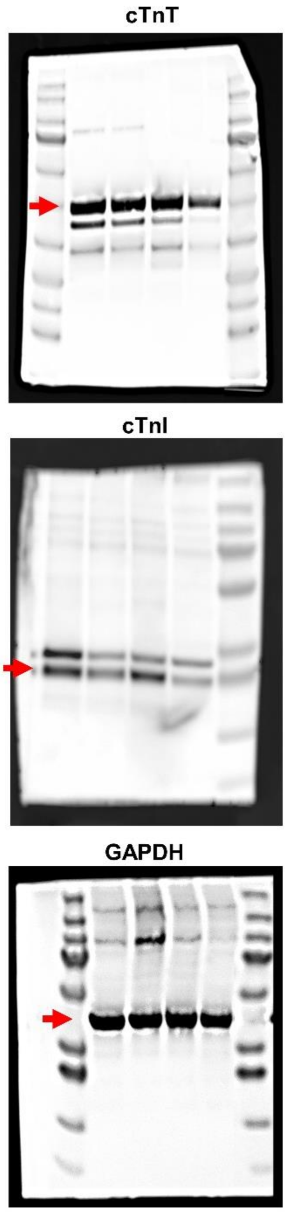

Supplementary figure 7. AMI and inflammation marker expression after IR injury. AMI and inflammation markers expression after IR injury. (A) Quantitative analysis of gene expression levels as performed with real-time PCR. The expression levels of inflammation markers (IL1α, IL1β, IL6, IL8, and COX2), necrosis marker (HMGB1) normalized to that of GAPDH. Data were shown as fold-change, as mean ± SD, by 2-way ANOVA (n=3 A significant difference of all graphs is indicated by $^{\#,*}p < 0.05$, $^{\#\#,**}p$ < 0.01 $^{\#\#\#,***}p < 0.001$, $^{\#\#\#\#,****}p < 0.0001$ (*Compared to control group; #compared to COs), ns (non-significant). (B) The protein expression of pNF-κB and NF-κB, which are immune modulation signaling pathways using western blot in cell lysates from COs and HOs in each group. (C) Representative western blot image and quantitative analysis of MAPK signaling (pp38, pERK, and pJNK) including inflammation, cell stress response. Quantitative analysis of all western blot data was calculated by the average intensity of the bands in Image J software. Equal protein loading amounts of western blot data were confirmed by GAPDH expression. A significant difference of all graphs is indicated by $^{\#,*}p < 0.05$, $^{\#\#,**}p < 0.01$ $^{\#\#\#,***}p < 0.001$, $^{\#\#\#\#,****}p < 0.0001$(*Compared to control group; #compared to COs), and ns (non-significant).

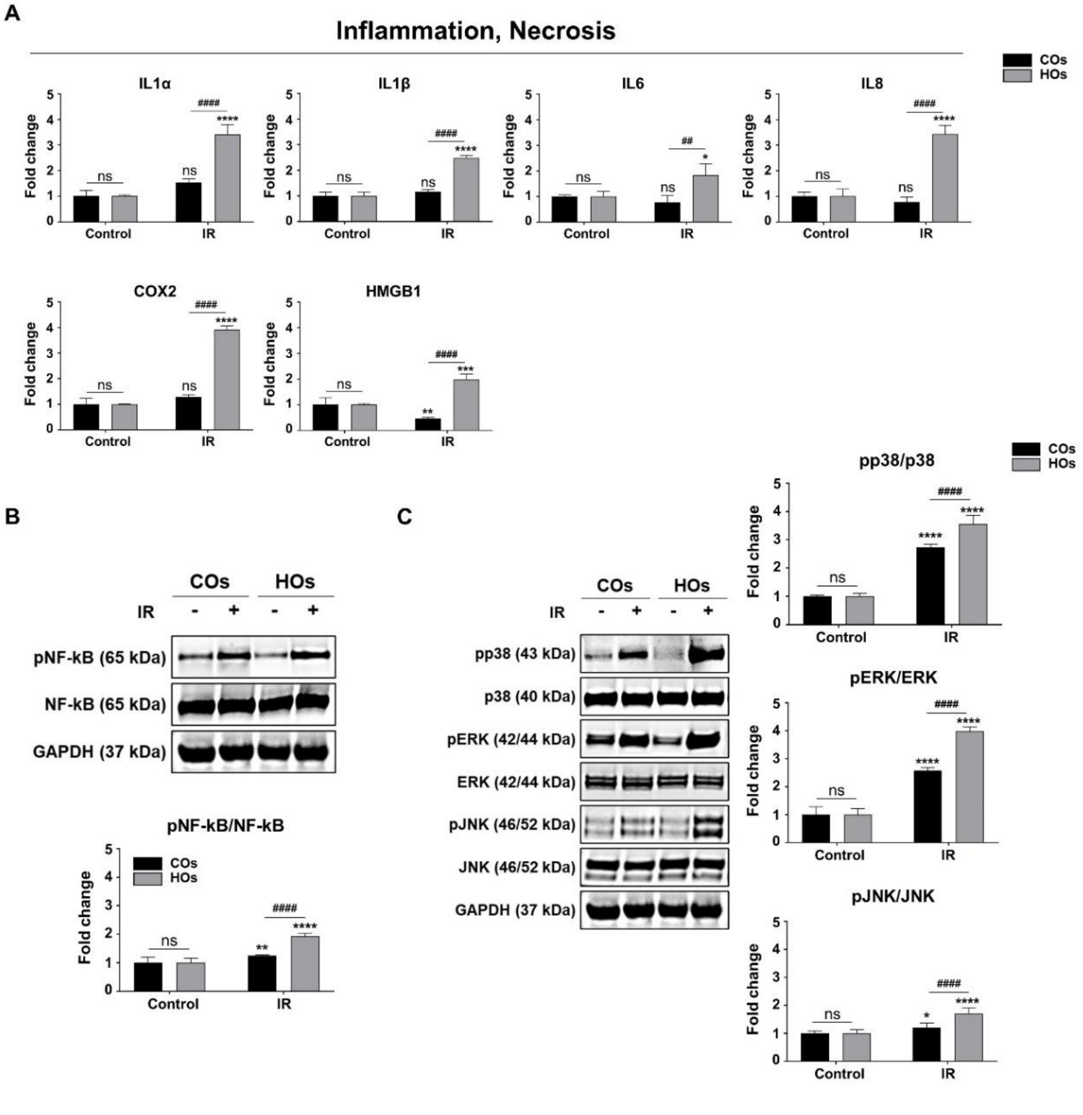

Supplementary figure 8. Original western blot images of Figure S7B and C. Bands in blue boxes were incorporated in this study. Another experimental condition was not described in this study.

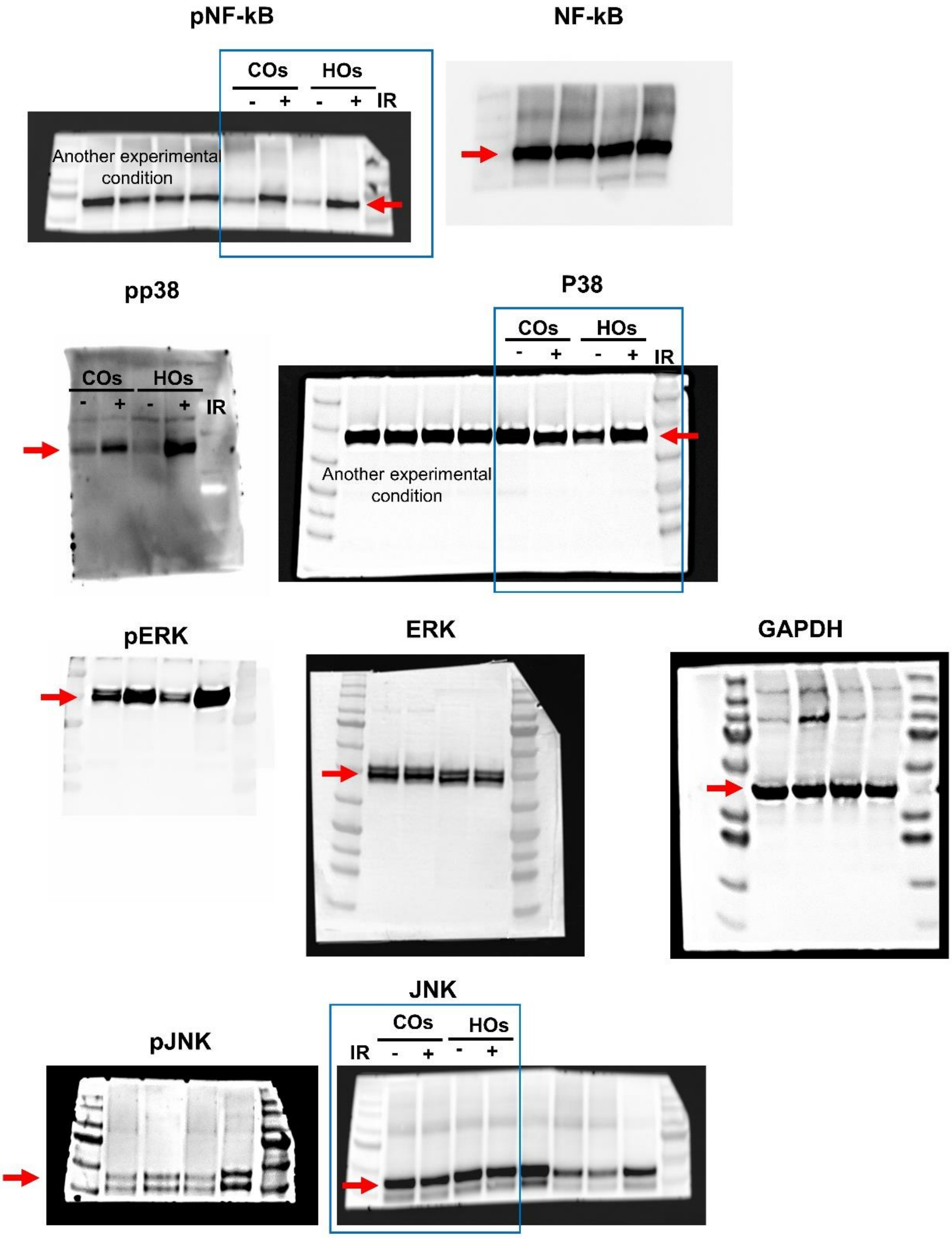

Supplementary figure 9. Original western blot images of Figure 6D. Bands in blue boxes were incorporated in this study.

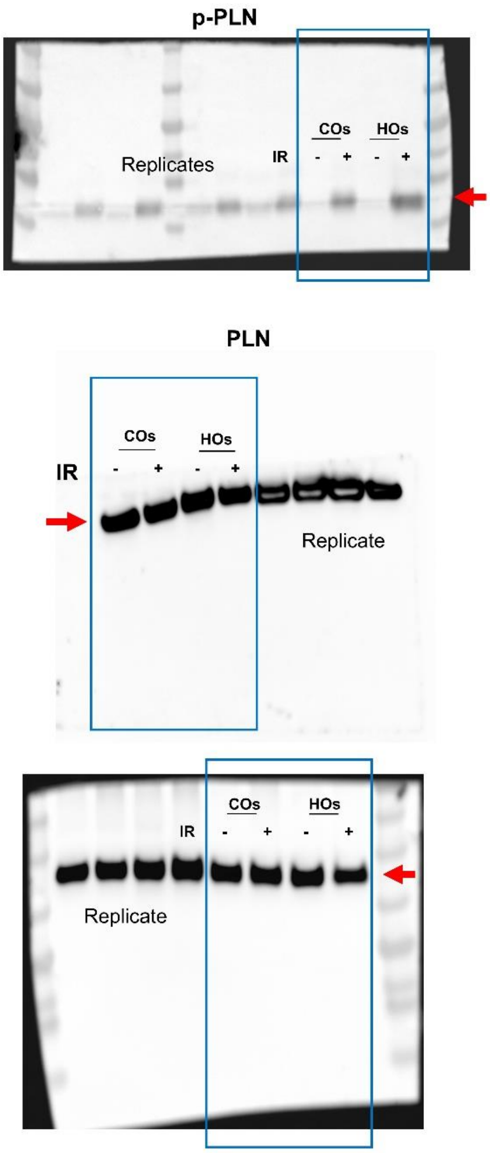

Supplement figure 10. Calcium transient assay after IR injury. Calcium transient assay after IR injury. Representative image of calcium transient COs and HOs in control, ischemia, and IR groups. The scale bar represents 200 μm.

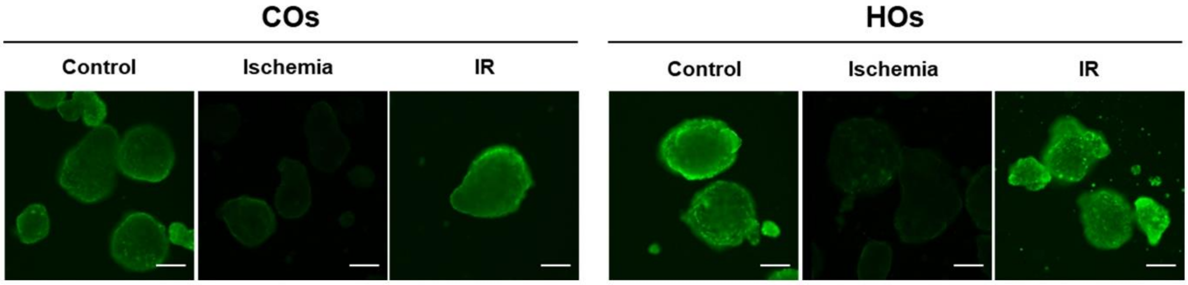

Supplement figure 11. Original WB image for Figure 7B. Bands in blue boxes were incorporated in this study. Another experimental condition was not described in this study.

COL1A1

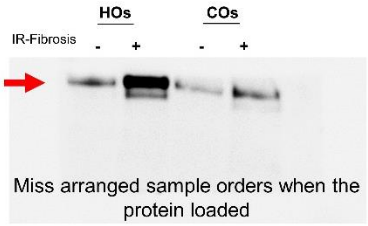

αSMA

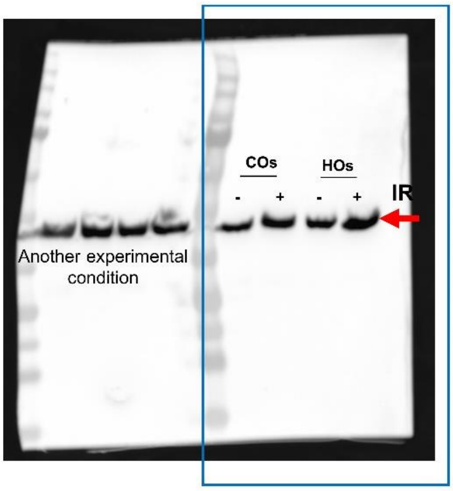

GAPDH

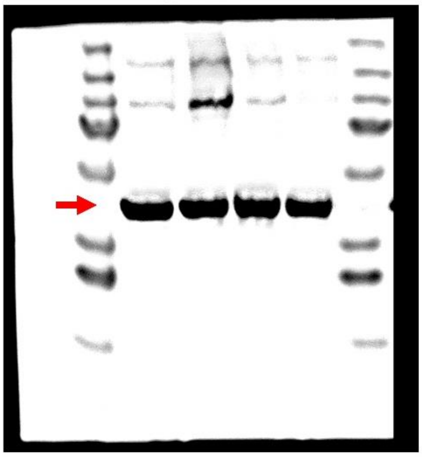

Supplement figure 12. Quantification of fibrosis and fibrosis-related gene expression in fibrosis-induced COs and HOs. (A) Quantitative analysis of COL1A1 immunofluorescence image (fig. 7A) performed using Image J. (B) Quantitative analysis of COL1A1 and αSMA expression by western blot data (fig. 7B) was calculated by the average intensity of the bands in Image J software. The comparison of the fold change between the groups was normalized by the control group. (C) The gene expression levels of fibrosis-related markers (ACTA2, POSTN, Vimentin, MMP2, and PAI1), collagen-related markers (COL1A1, COL1A2, and COL3A1) of COs and HOs in IR-Fibrosis group. These genes normalized to that of GAPDH. All data were shown as fold-change relative to control group, as mean ± SD, by 2-way ANOVA (n=3). A significant difference of all graphs is indicated by $^{\#,*}p < 0.05$, $^{\#\#,**}p < 0.01$ $^{\#\#\#,***}p < 0.001$, $^{\#\#\#\#,****}p < 0.0001$(*Compared to control group; #compared to COs) and ns (non-significant).

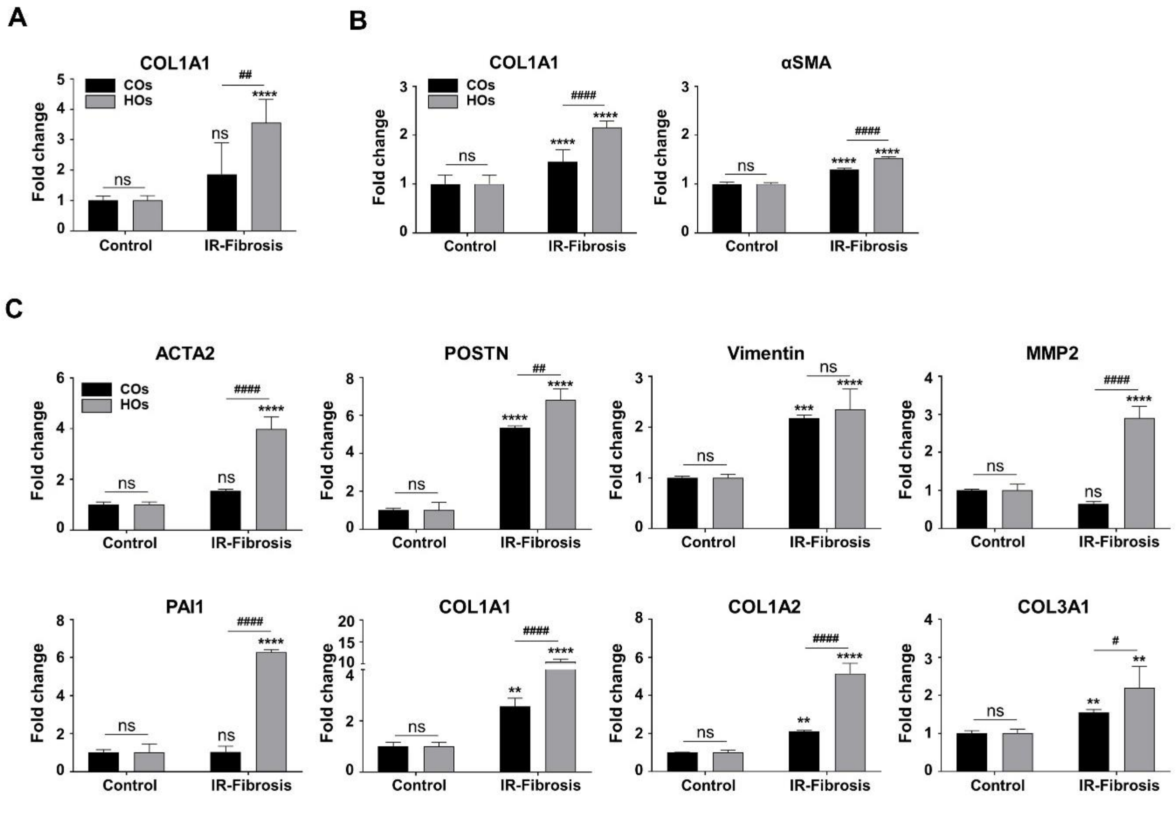

Supplement figure 13. Calcium transient assay in fibrosis-induced COs and HOs. (A) Representative image of calcium transient COs and HOs in control and IR-Fibrosis groups. The scale bar represents 200 μm. (B) Beating characteristics of COs and HOs in each group. Beating analysis was performed by monitoring calcium fluorescence over a period of 20 seconds under each condition.

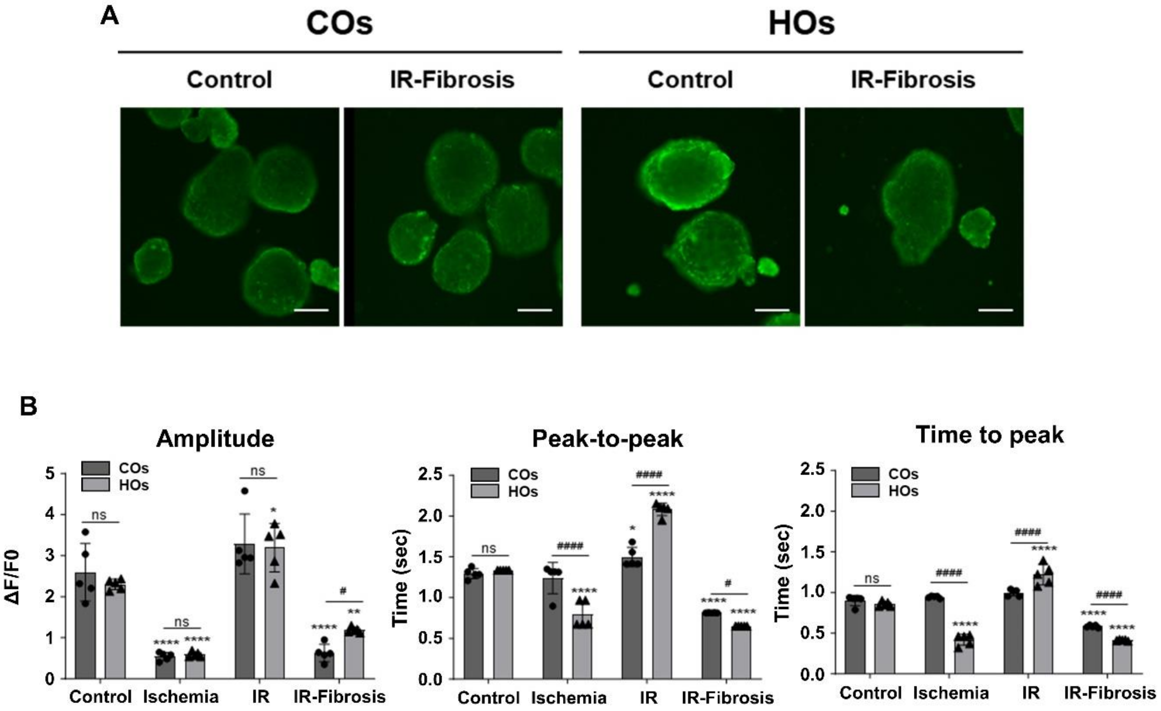

Supplementary Tables

Supplementary Table 1. List of antibodies used in flowcytometry analysis.

Antibody	Cat.no	Host	Dilution
Mouse IgG	sc-3877	Mouse	1:40
cTnT	ab8295	Mouse	1:400
VE-CAD	sc-9989	Mouse	1:40
CD90	14-0909-82	Mouse	1:100
Rabbit IgG	ab171870	Rabbit	1:1,000
CD31	11265-1-AP	Rabbit	1:400
Phosphlamban	A010-14	Mouse	1:5000

Supplementary Table 2. List of primers used in q-PCR analysis.

Gene	Forward	Reverse
GAPDH	5′-GATGACATCAAGAAGGTGGTGA-3′	5′-TTCGTTGTCATACCAGGAAATG-3′
NKX2.5	5′-CCTACGGTTATAACGCCTACCC-3′	5′-GCTCTGAACCGCATTCAAGT-3′
TNNT2	5′-AAGAGGCAGACTGAGCGGGAAA-3′	5′-AGATGCTCTGCCACAGCTCCTT-3′
MYL2	5′-CGGAGAGGTTTTCCAAGGAGGA-3′	5′-CTCTTCTCCGTGGGTGATGATG-3′
MYL7	5′-CCGTCTTCCTCACGCTCTTTG-3′	5′-TGAACTCATCCTTGTTCACCACC-3′
CD34	5′-CAGTGTCTACTGCTGGTCTTGG-3′	5′-CAATCAGGGTCTTTTGGGAATA-3′
PECAM1	5′-TCTGAACTCCAACAACGAGAAA-3′	5′-CACTTGAACTTCCGTGTACTGC-3′
SOX17	5′-CGCACGGAATTTGAACAGTAT-3′	5′-CACACGTCAGGATAGTTGCAGT-3′
FOXA2	5′-CCCACAAAATGGACCTCAAG-3′	5′-AGTACACCCCCTGGTAGTAGGA-3′
CD90	5′-CAACTTCACCAGCAAATACAACAT-3′	5′-CTCACACTTGACCAGTTTGTCTCT-3′
PDGFRA	5′-CTCCTTTTACCACCTGAGTGAGATT-3′	5′-TTTTTGTAGGTGACACCAATGTATG-3′
Vimentin	5′-TGAGATTCAGAATATGAAGGAGGAA-3′	5′-AGAGGGAGTGAATCCAGATTAGTTT-3′
TCF21	5′-AAGGCCTTCTCCAGACTCAAG-3′	5′-GTGAATGTACCCGTTCTCGTATTT-3′
IL1A	5′-TCTGAGTGTGACCAGGCATC-3′	5′-TGGACCAAAATGCCCTGTAT-3′
IL1B	5′-GCATCCAGCTACGAATCTCC-3′	5′-GCATCTTCCTCAGCTTGTCC-3′
IL6	5′-ACTCACCTCTTCAGAACGAATTG-3′	5′-CCATCTTTGGAAGGTTCAGGTTG-3′
IL8	5′-GAGAGTGATTGAGAGTGGACCAC-3′	5′-CACAACCCTCTGCACCCAGTTT-3′
COX2	5′-GCAATAACGTGAAGGGCTGT-3′	5′-CGGGAAGAACTTGCATTGAT-3′
HMGB1	5′-AGGGAGTTGTCAAGGCTGAA-3′	5′-CCTCCTCCTCCTCATCCTCT-3′
ACTA2	5′-CTATGCCTCTGGACGCACAACT-3′	5′-CAGATCCAGACGCATGATGGCA-3′
POSTN	5′-CATGCATGGAGAAAGGGAGT-3′	5′-TGCAGCTTCAAGTAGGCTGA-3′
MMP2	5′-ACGACCGCGACAAGAAGTAT-3′	5′-ATTTGTTGCCCAGGAAAGTG-3′
PAI1	5′-CTCATCAGCCACTGGAAAGGCA-3′	5′-GACTCGTGAAGTCAGCCTGAAAC-3′
COL1A1	5′-GTGCTAAAGGTGCCAATGGT-3′	5′-ACCAGGTTCACCGCTGTTAC-3′
COL1A2	5′-CCTGGTGCTAAAGGAGAAAGAGG-3′	5′-ATCACCACGACTTCCAGCAGGA-3′
COL3A1	5′-TGGTCTGCAAGGAATGCCTGGA-3′	5′-TCTTTCCCTGGGACACCATCAG-3′

Supplementary references

1. Lee H, Im JS, Choi DB, An J, Kim S-B, Yeon S, et al. Three-dimensional cardiac organoid formation accelerates the functional maturation of human induced pluripotent stem cell-derived cardiomyocytes. Organoid. 2022;2.

2. Sala L, van Meer BJ, Tertoolen LGJ, Bakkers J, Bellin M, Davis RP, et al. MUSCLEMOTION: A Versatile Open Software Tool to Quantify Cardiomyocyte and Cardiac Muscle Contraction In Vitro and In Vivo. Circ Res. 2018;122(3):e5-e16.

3. Dobin A, Davis CA, Schlesinger F, Drenkow J, Zaleski C, Jha S, et al. STAR: ultrafast universal RNA-seq aligner. Bioinformatics. 2013;29(1):15-21.

4. Anders S, Pyl PT, Huber W. HTSeq--a Python framework to work with high-throughput sequencing data. Bioinformatics. 2015;31(2):166-9.
